# Synthesis, Optical Properties, and Antiproliferative Evaluation of NBD-Triterpene Fluorescent Probes

**DOI:** 10.1021/acs.jnatprod.2c00880

**Published:** 2022-12-21

**Authors:** Marta Medina-O’Donnell, Karina Vega-Granados, Antonio Martinez, M. Rosario Sepúlveda, José Antonio Molina-Bolívar, Luis Álvarez de Cienfuegos, Andres Parra, Fernando J. Reyes-Zurita, Francisco Rivas

**Affiliations:** ^†^Departamento de Química Orgánica, ^‡^Departamento de Biología Celular, and ^§^Departamento de Bioquímica y Biología Molecular I. Facultad de Ciencias, Universidad de Granada, E-18071Granada, Spain; ∥ Departamento de Física Aplicada II. Escuela de Ingeniería, Universidad de Málaga, E-29071Málaga, Spain

## Abstract

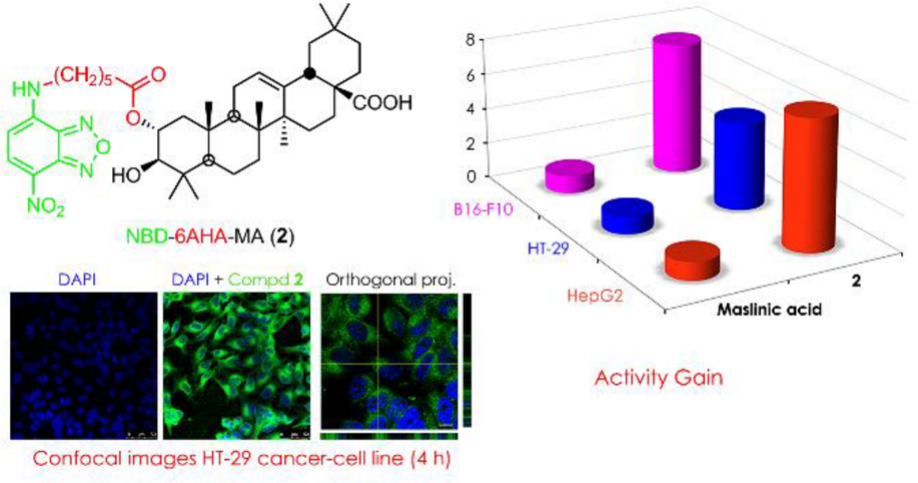

A fluorescent labeling protocol for hydroxylated natural compounds with promising antitumor properties has been used to synthesize, in yields of 72–86%, 12 derivatives having fluorescent properties and biological activity. The reagent used for the synthesis of these fluorescent derivatives was 7-nitrobenzo-2-oxa-1,3-diazole chloride (NBD-Cl). The linkers employed to bind the NBD-Cl reagent to the natural compounds were ω-amino acids (Aa) of different chain lengths. The natural triterpene compounds chosen were oleanolic and maslinic acid, as their corresponding 28-benzylated derivatives. Thus, 12 NBD-Aa-triterpene conjugates have been studied for their optical fluorescence properties and their biological activities against cell proliferation in three cancer cell lines (B16-F10, HT-29, and HepG2), compared with three nontumor cell lines (HPF, IEC-18, and WRL68) from different tissues. The results of the fluorescence study have shown that the best fluorescent labels are those in which the ω-amino acid chain is shorter, and the carboxylic group is not benzylated. Analysis by confocal microscopy showed that these compounds were rapidly incorporated into cells in all three cancer cell lines, with these same derivatives showing the highest toxicity against the cancer cell lines tested. Then, the fluorescent labeling of these NBD-Aa-triterpene conjugates enabled their uptake and subcellular distribution to be followed in order to probe in detail their biological properties at the cellular and molecular level.

Natural products continue to be a great source of pharmacologically active compounds that are used as a starting point for new drug discovery.^1−3^ Although this percentage has decreased recently, natural products continue to play an important role in drug discovery. For example, in the field of anticancer compounds, over the last four decades, of the 185 small molecules approved, one-third (62) are related to natural products or their derivatives.^4,5^ Analogues of natural products, obtained through chemical processes based on rational designs, which tend to have higher efficiency, higher toxicity and better bioavailability than their precursors, are widely used today.^6−8^

Natural pentacyclic triterpenes are the most biologically attractive triterpenoids. These natural compounds are widely distributed in plants and other living organisms, presenting a wide spectrum of biological properties, such as antiviral,^9,10^ antibacterial,^11,12^ anti-inflammatory,^13,14^ and antitumor^15,16^ activity. Among the pentacyclic triterpenes with oleanane skeleton, oleanolic acid^17^ and maslinic acid^18^ are two of the most widely investigated in the past decade, due to their biological activities.^19−21^ Both triterpenoid acids are found in large quantities in olive-mill wastes, from which they are efficiently extracted using various methods.^22,23^ Recently, this abundant isolated raw material has enabled us to perform the semisynthesis of different conjugates of the two triterpenoid acids, with promising biological properties as antitumor or anti-HIV agents.^24−26^ The semisynthetic derivatives of the pentacyclic triterpenes, properly conjugated with other molecules, have great potential as anticancer or antiviral agents, and have even been used in studies at the molecular level.^27^

Fluorescence microscopy is a particularly important technique in the field of biochemistry and cell imaging to analyze the uptake and subcellular distribution of fluorescent compounds and to study their involvement in cellular processes.^28,29^ Thus, the exploration of a fast, efficient, and inexpensive fluorescent labeling protocol for bioactive natural products facilitates the investigation of cellular events. Recently, much progress has been made in the development of methods to identify cell targets or determine the mode of action of small molecules, with the help of fluorescent probes.^30,31^

Widely varying natural fluorescent compounds have been used as probes for different biological studies.^32^ However, only a few recent studies have been conducted using pentacyclic triterpenes conjugated to fluorophore groups.^33−37^ Derivatives of oxadiazole have also been used to form these fluorescent probes. In particular, 7-nitrobenzo-2-oxa-1,3-diazole chloride (NBD-Cl) is a nonfluorescent molecule that is nevertheless capable of undergoing nucleophilic substitution through an amino group, and therefore becomes a sensitive fluorescent entity.^38^ Other examples of labeling natural products and bioorganic compounds using NBD-Cl as a reagent to obtain fluorescence have also been studied.^39−41^

In the present work, the NBD-Cl reagent has been coupled to oleanolic acid (**OA**), through the C-3 hydroxy group, or maslinic acid (**MA**), through the C-2 hydroxy group of ring A of the triterpene skeleton, using various linkers to form different fluorescent probes. The linkers used were ω-amino acids of different chain lengths, such as 6-aminohexanoic acid (A6AH), 8-aminooctanoic acid (A8AO), and 11-aminoundecanoic acid (A11AU). Analogous fluorescent probes were also synthesized from the corresponding 28-benzyl derivatives (28-benzyl oleanolate, **BO**; and 28-benzyl maslinate, **BM**). The 12 NBD-Aa-triterpene fluorescent probes synthesized were studied for their chemical, spectroscopic, and fluorescent characteristics, as well as their biological activity as tested in different cell lines.

## Results and Discussion

### Chemistry

**Figure 1:**
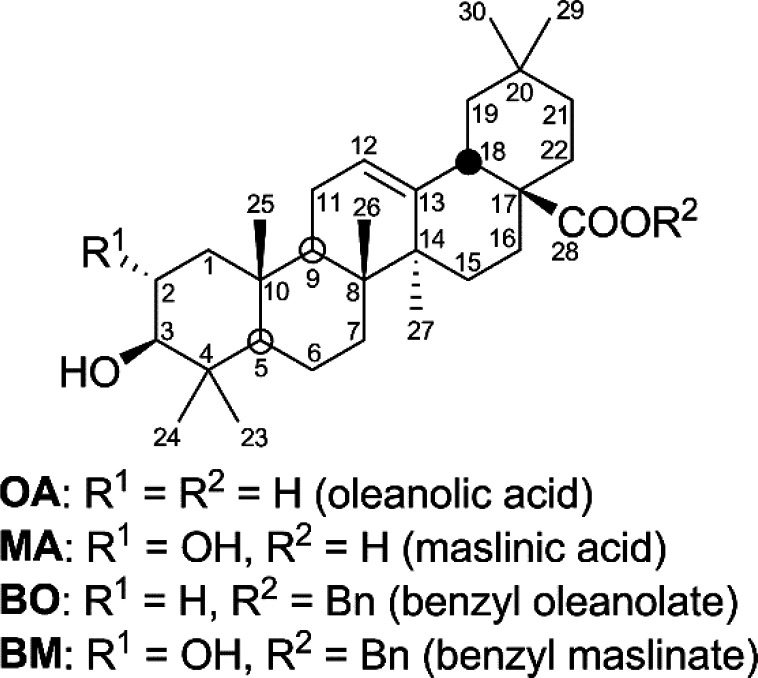
Structures of the triterpene compounds used.

Different fluorescent derivatives of triterpenoid compounds were synthesized according to a protocol in a single flask (one-pot).^38^ The triterpenes used were oleanolic acid (3β-hydroxyolean-12-en-28-oic acid, **OA**) and maslinic acid (2α,3β-dihydroxyolean-12-en-28-oic acid, **MA**), present in olive-mill wastes, and their corresponding 28-benzylated derivatives, such as 28-benzyl oleanolate (**BO**) and 28-benzyl maslinate (**BM**) (Figure 1).

**Scheme 1:**
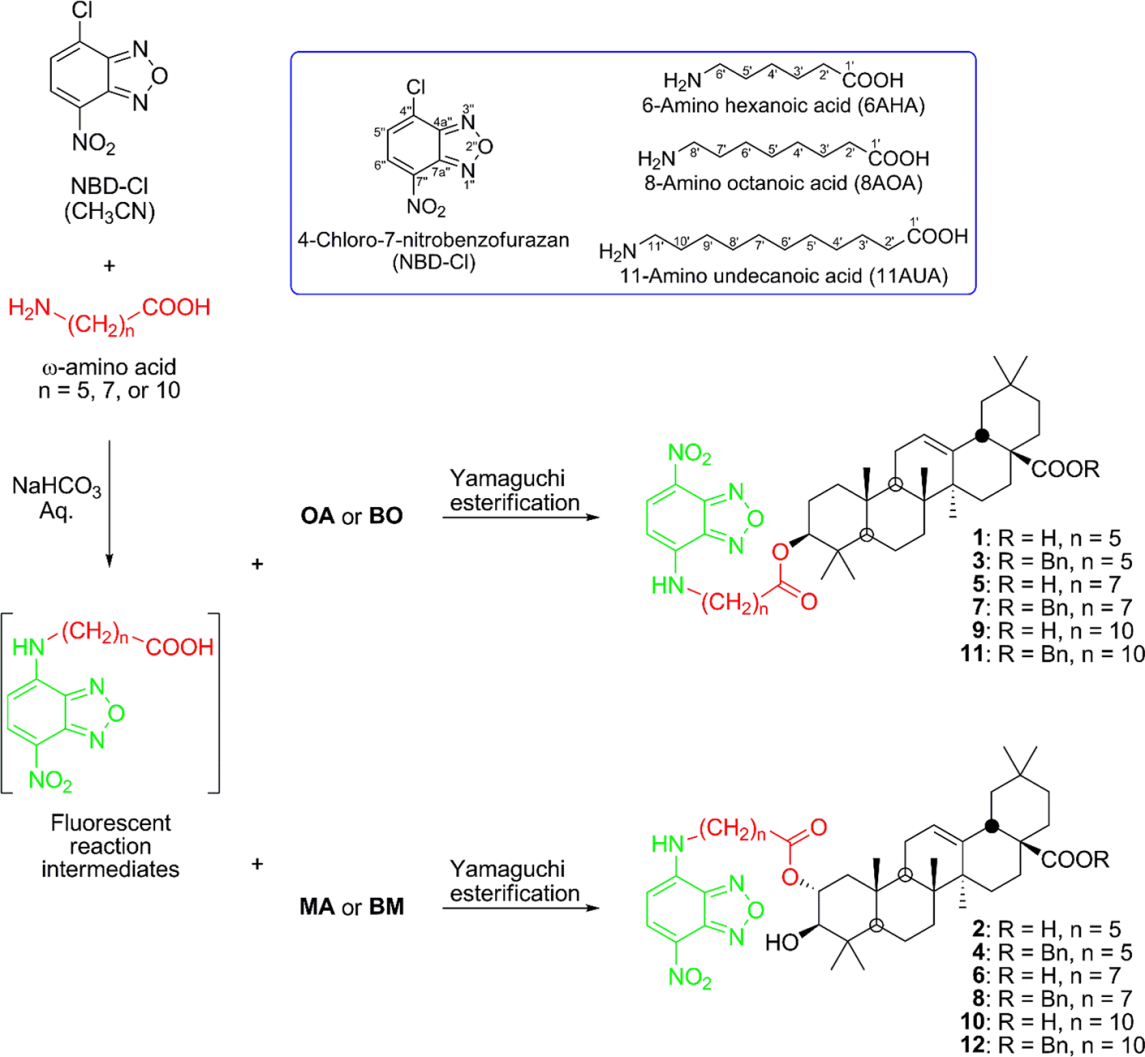
One-Pot Synthesis of Fluorescent Triterpene Conjugates **1**–**12**

The reagent used to form the fluorescent compounds was 4-chloro-7-nitrobenzofurazan (NBD-Cl). This NBD-Cl was attached to a ω-amino acid of different chain lengths, specifically 6-aminohexanoic acid (6AHA), 8-aminooctanoic acid (8AOA), and 11-aminoundecanoic acid (11AUA). The appropriate conjugation reaction (see Experimental Section) of the corresponding fluorescent reaction intermediate with each of the four triterpenoid compounds (**OA**, **MA**, **BO**, or **BM**), resulted in the formation of 12 NBD-Aa-triterpene conjugates (Scheme 1).

According to the one-pot protocol, NBD-Cl was treated with 6AHA and **OA** successively without purifying the intermediates, thus providing the NBD-6AHA-**OA** conjugate (**1**). This derivative (**1**) exhibited a molecular formula of C_42_H_60_N_4_O_7_ that corresponded to a molar mass of 732 Da, evidencing the nucleophilic substitution of NBD-Cl by 6AHA and the subsequent esterification of the resulting carboxylic acid with **OA**. In the ^1^H NMR spectrum of derivative **1**, the signal from the geminal proton to the ester group at C-3 (δ_H_ 4.51, 1H, dd, *J* = 8.0, 8.0 Hz), and in its ^13^C NMR spectrum, the C-3 signal (δ_C_ 81.2), confirmed this esterification reaction between the hydroxy group at C-3 of **OA** and the carboxylic acid group of 6AHA.

Derivative **2**, NBD-6AHA-MA, obtained by reacting NBD-Cl with 6AHA and **MA**, successively, gave a molar mass of 748 Da and a molecular formula of C_42_H_60_N_4_O_8_. The ^1^H and ^13^C NMR signals of this derivative, **2**, were similar to those of derivative **1**. The only difference between the two derivatives (**1** and **2**) is that maslinic acid has two hydroxy groups at C-2 and C-3, making it necessary to determine which OH group was esterified with the intermediate NBD-6AHA. Thus, in the ^1^H NMR spectrum of derivative **2**, the H-2 signal appeared at δ_H_ 5.01, more deshielded than in **MA** (3.62 ppm),^42^ indicating that esterification occurred at the C-2 hydroxy group. The position of this esterification was also confirmed by analyzing the ^13^C NMR data for C-2 (73.2 ppm for derivative **2** and 69.5 ppm for **MA**).^42^ This esterification occurred in only one of the hydroxy groups, due to the use of stoichiometric amounts for the reactions of maslinic acid, occurring on the hydroxy group of C-2 because the OH group at C-3 was more hindered, due to the presence of the *gem*-dimethyl group located in the contiguous position of C-4.

Conjugated derivatives **3** and **4** were derived from the reaction of NBD-Cl, 6AHA, and 28-benzyl oleanolate (**BO**) or 28-benzyl maslinate (**BM**). These compounds showed ^1^H and ^13^C NMR signals similar to those of derivatives **1** and **2**, respectively, except for the signals of the benzyl group on the C-28 carboxylic group.

Conjugated derivatives **5**–**8** were prepared similarly to derivatives **1**–**4**, but now using a longer-chain ω-amino acid, such as 8-aminooctanoic acid (8AOA), as the linker. Finally, conjugated derivatives **9**–**12** were formed using the same one-pot protocol but using an even longer-chain ω-amino acid, such as 11-aminoundecanoic acid (11AUA), as the linker. The signals of the ^1^H and ^13^C NMR spectra of these compounds (**5**–**12**) were consistent with the proposed structures.

### Optical Properties

**Figure 2:**
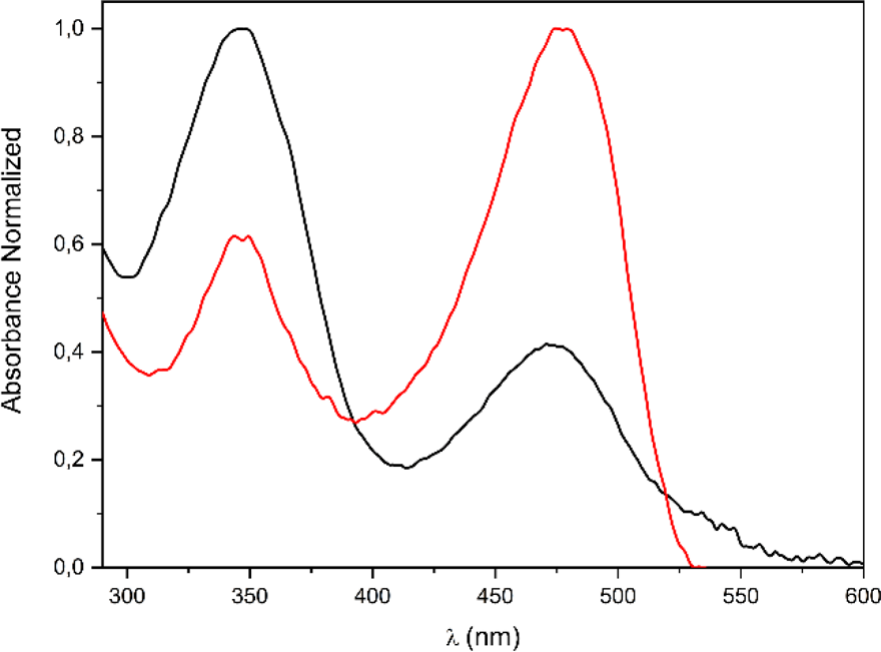
Normalized absorption spectra of NBD-Cl (black) and conjugate **1** (red).

The optical properties of the 12 fluorescent derivatives, obtained from NBD-Cl, an ω-amino acid, and triterpenes **OA**, **MA**, **BO**, or **BM** (**1**–**12**), were studied by absorption and fluorescence spectroscopy. The normalized absorption spectra of reagent NBD-Cl and conjugate **1**, were analyzed. The spectra of the rest of the derivatives (**2**–**12**) were shown to be similar. Conjugate **1** exhibited the longest wavelength absorption band in the 400–520 nm range, with well-pronounced maxima at approximately 480 nm. This maximum was less noticeable for NBD-Cl and appeared at 471 nm, exhibiting the longest wavelength absorption band in the 300–400 nm range (Figure 2).

The values of the maximum absorption wavelength (λ_abs_), the fluorescence emission wavelength (λ_em_), the molar extinction coefficient (ε), the fluorescence quantum yield (Φ_F_), and the half-life time of fluorescence (τ) are tabulated in Table 1.

**Table 1:** Photophysical Absorption and Fluorescence Data of NBD-Cl and Compounds **1**–**12**

triterpene compounds	fluorescent triterpene conjugates	(λ_abs_)max (nm)	(λ_em_)max (nm)	ε (M^–1^ cm^–1^)	Φ	τ (ns)
	NBD-Cl	471	557	369		
**OA** conjugates	**1**	480	546	2539	0.76	7.712
	**5**	480	545	1144	0.73	7.654
	**9**	481	546	1015	0.71	7.635
**MA** conjugates	**2**	480	546	3166	0.71	7.681
	**6**	481	546	1986	0.55	7.621
	**10**	481	546	1199	0.45	7.153
**BO** conjugates	**3**	480	545	4035	0.67	7.761
	**7**	480	547	2645	0.71	7.724
	**11**	480	546	2718	0.49	7.645
**BM** conjugates	**4**	480	545	1338	0.72	7.650
	**8**	481	548	3358	0.59	7.639
	**12**	480	546	2710	0.46	7.633

The effect of the length of the amino acid chain influenced the optical properties of these derivatives (**1**–**12**). The length of the ω-amino acid chain did not affect the absorption and emission maxima of these derivatives. However, the half-life time of fluorescence (τ) and, in most cases, the fluorescence quantum yield (Φ_F_) decreased with increasing amino acid chain length. The molar extinction coefficient (ε), in **OA** or **MA** conjugates (**1**, **2**, **5**, **6**, **9**, and **10**), decreased with increasing length of the amino acid chain, while, in **BO** and **BM** conjugates (**3**, **4**, **7**, **8**, **11**, and **12**), the influence of the chain length of amino acids on the molar extinction coefficient (ε) was unclear (Table 1).

**Figure 3:**
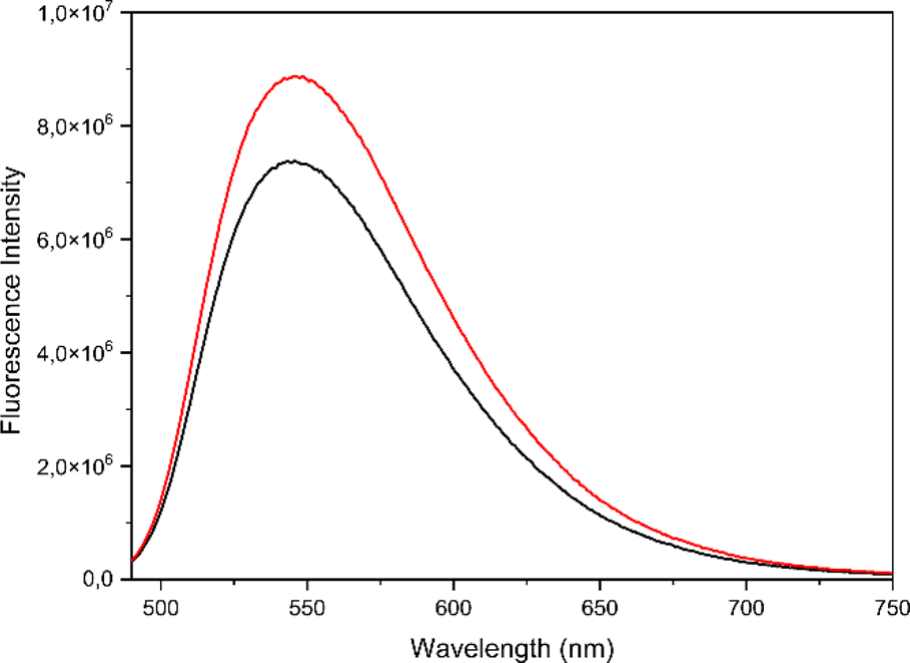
Fluorescence spectra of conjugates **1** (black) and **2** (red) at 30 μM in DMSO.

Figure 3 displays the fluorescence spectra of derivatives **1** and **2** in DMSO solution. **MA** conjugate **2** was more fluorescent than **OA** conjugate **1**. The same trend was observed for the rest of the **OA** and **MA** conjugates.

**Figure 4:**
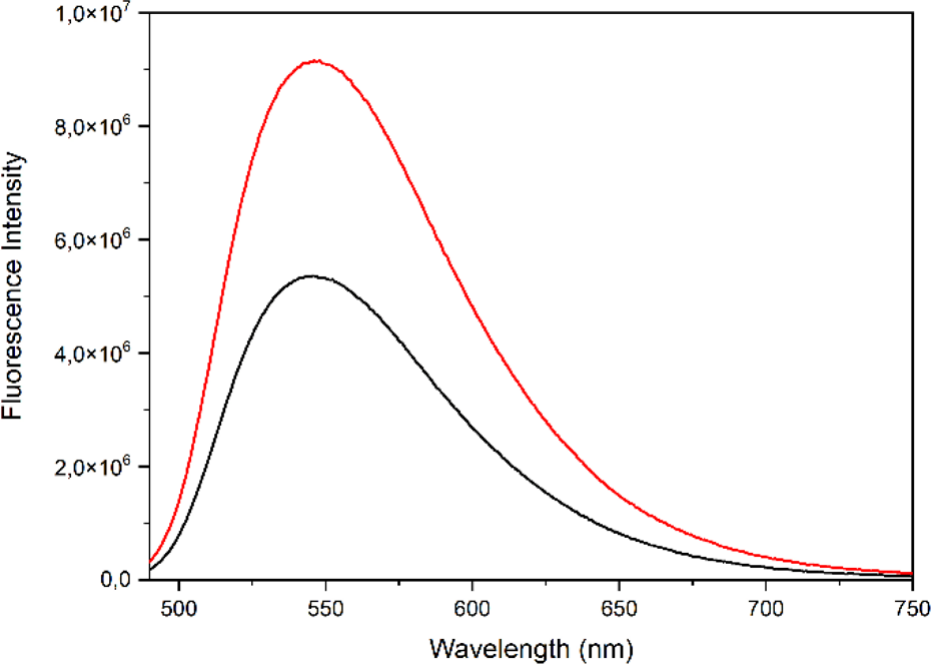
Fluorescence spectra of conjugates **6** (red) and **10** (black) at 56 μM in DMSO.

The fluorescence of these derivatives (**1**–**12**) did not improve with greater length of the ω-amino acid chain. This increase in length decreased the quantum yield, which reduced the fluorescence efficiency of these conjugated triterpenes. A decrease in the half-life time was also observed, which implies more abrupt decay of the fluorescence of these derivatives. For example, **MA** conjugate **6** was more fluorescent than **MA** conjugate **10**, with a longer amino acid chain (Figure 4).

### Effects of NBD-Aa-Triterpene Conjugates on Cancer Cell Viability

The cytotoxic effects of 12 NBD-Aa-triterpene conjugates (**1**–**12**) were tested on three different cancer cell lines as a representation of cancers from different tissues and organs (B16-F10 murine melanoma, HT-29 human colon adenocarcinoma, and HepG2 human hepatocyte carcinoma). Cell viability was determined by MTT colorimetric analysis, with increasing concentration levels of each compound (0–200 μg/mL) and incubation for 72 h. The MTT assay was based on the metabolic reduction of 3-(4,5-dimethylthiazol-2-yl)-2,5-diphenyltetrazolium bromide (MTT) to a violet-colored compound (formazan), with a maximum absorbance at 570 nm. Cell viability was proportional to the amount of formazan and was expressed as the percentage of viable treated cells relative to untreated control cells. The concentration at which the derivatives halved the number of viable cells in the three cancer lines (IC_50_) was determined. NBD conjugates **1** and **2**, with the shortest-chain ω-amino acid linker (6AHA), showed the most potent cytotoxic results. The IC_50_ concentrations of these derivatives (**1** and **2**) were less than 10 μM or, in any case, did not exceed 20 μM (Table 2). These conjugates were between 44- and 5-fold more effective than their corresponding precursors (**OA** or **MA**). In NBD conjugates **5**, **6**, **9**, and **10**, with longer-chain ω-amino acid linkers (8AOA or 11AUA), the IC_50_ concentrations were much higher, above 50 μM in most cases, which also occurred for the NBD conjugates of **BO** or **BM** (**3**, **4**, **7**, **8**, **11**, and **12**), which reached IC_50_ values greater than 80 μM in most cases. These NBD conjugates of **BO** or **BM** also had higher IC_50_ values than their precursors (**BO** or **BM**), even in NBD conjugates with the shortest-chain ω-amino acid linker (6AHA) (Table 2). Due to these high IC_50_ values, all of these NBD-conjugated compounds were discarded for further cytometric studies.

**Table 2:** IC_50_ Values of NBD-Aa-Triterpene Conjugates **1**–**12** on Three Cancer Cell Lines

compound	B16-F10^a^	HT-29^a^	HepG2^a^
**OA**	106.4 ± 3.7	429.9 ± 0.7	211.8 ± 0.5
**MA**	36.2 ± 2.5	32.2 ± 3.8	99.2 ± 5.5
**BO**	52.2 ± 0.9	67.1 ± 3.2	38.7 ± 1.7
**BM**	19.0 ± 0.2	15.3 ± 0.5	17.0 ± 0.0
**1**	2.4 ± 0.1	19.5 ± 0.1	12.5 ± 0.1
**2**	4.9 ± 0.4	6.6 ± 0.1	13.9 ± 0.6
**3**	76.6 ± 3.7	63.3 ± 2.4	74.3 ± 0.7
**4**	96.4 ± 0.9	120.7 ± 4.0	91.2 ± 2.3
**5**	136.8 ± 2.0	105.4 ± 1.2	95.7 ± 3.4
**6**	39.5 ± 0.8	45.2 ± 0.9	98.5 ± 0.8
**7**	74.2 ± 2.2	95.9 ± 2.2	71.6 ± 1.7
**8**	96.8 ± 1.8	122.8 ± 1.7	95.3 ± 0.7
**9**	101.1 ± 0.9	113.0 ± 4.4	84.5 ± 0.4
**10**	107.7 ± 0.4	109.8 ± 5.2	122.8 ± 0.9
**11**	92.3 ± 1.5	100.9 ± 1.1	82.7 ± 2.5
**12**	80.8 ± 0.9	108.3 ± 3.8	80.9 ± 1.1

a The IC_50_ values (μM) were calculated by considering untreated control cells as 100% viability. Cell viability was analyzed using the MTT assay, as described in the Experimental Section.

Due to their greater cytotoxic activity, NBD conjugates **1** and **2** were selected to analyze their cytotoxic effects on healthy cells, using nontumor cell lines (HPF human pulmonary fibroblasts, IEC-18 epithelial rat ileum cells, and WRL68 human embryonic hepatic cells) (Table 3). The selectivity indices (SI) of the NBD-Aa-triterpene conjugates **1** and **2** were calculated with the formula: IC_50_ nontumor cells/IC_50_ cancer cells. Additionally, cell-viability percentages in the nontumor cells were measured, which ranged in most cases between 75 and 90%.

**Table 3:** Cytotoxic Effects of NBD-6AHA-Triterpene Conjugates **1** and **2** on Nontumor Cells

compound	HPF^a^	SI^b^	IEC-18^a^	SI^b^	WRL68^a^	SI^b^
**1**	39.7 ± 1.2	17	41.0 ± 0.3	2	37.9 ± 0.2	3
**2**	14.8 ± 1.5	3	14.7 ± 0.7	2	10.0 ± 0.3	1

a The IC_50_ values (μM) were calculated by considering untreated control cells as 100% viability. Cell viability was analyzed using the MTT assay, as described in the Experimental Section.

b SI = selectivity index (IC_50_ nontumor cells/IC_50_ cancer cells).

### Flow-Cytometry Characterization of Cell Death

NBD-6AHA-triterpene conjugates **1** and **2** were selected for the characterization of their cytotoxic effects on the three cancer cell lines used. The percentages of cell death were determined using the concentrations corresponding to the IC_50_ values for each compound in the three cancer cell lines (B16-F10, HT-29, and HepG2). This study was conducted by flow cytometry, using double staining with DY634 annexin V/propidium iodide, which allows the identification and quantification of intact, apoptotic, or necrotic cells. Annexin DY634 (red fluorescence) is an apoptotic marker due to its ability to bind to phosphatidylserine exposed on the external cell surface. Propidium iodide (orange fluorescence) is a DNA marker that is capable of penetrating only when the cell is damaged, differentiating late apoptotic or necrotic cells from apoptotic ones. These conjugates (**1** and **2**) are green fluorescent probes, not causing interference in the fluorescence.

**Figure 5:**
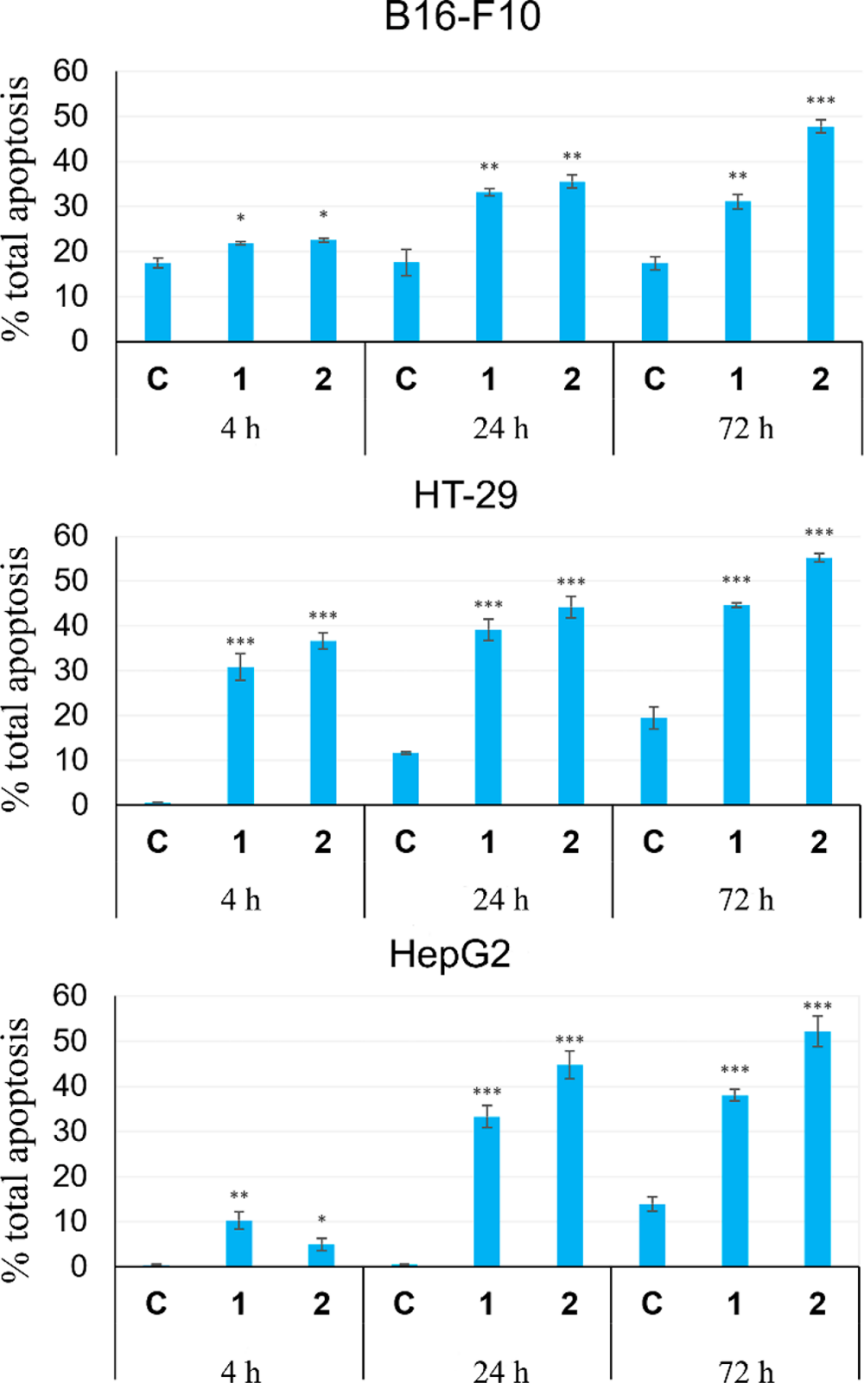
Cell death analysis by flow cytometry after exposure of B16-F10, HT-29, and HepG2 cells to conjugates **1** and **2** and control. Cell lines were treated at concentrations corresponding to IC_50_ values for 4, 24, and 72 h. Data are expressed as means ± SEM of at least two experiments in triplicate. *p* < 0.05 (*), *p* ≤ 0.01 (**), and *p* ≤ 0.001 (***), compared to untreated-control cells.

The apoptosis studies were conducted at 4, 24, and 72 h (Figure 5). These two derivatives showed apoptotic effects in the treated cells, with this effect being significant after 24 h and especially after 72 h, having percentages of total apoptosis between 31 and 55% in the three tumor cell lines. The NBD conjugate of **MA** (**2**) showed greater apoptotic effects than did the NBD conjugate of OA (**1**), in all three cancer cell lines, with percentages of apoptosis after 72 h of 48% for the B16-F10 line, 55% for the HT-29 line, and 53% for the HepG2 line.

### Analysis of the Cellular Uptake of Compounds by Confocal Microscopy

**Figure 6:**
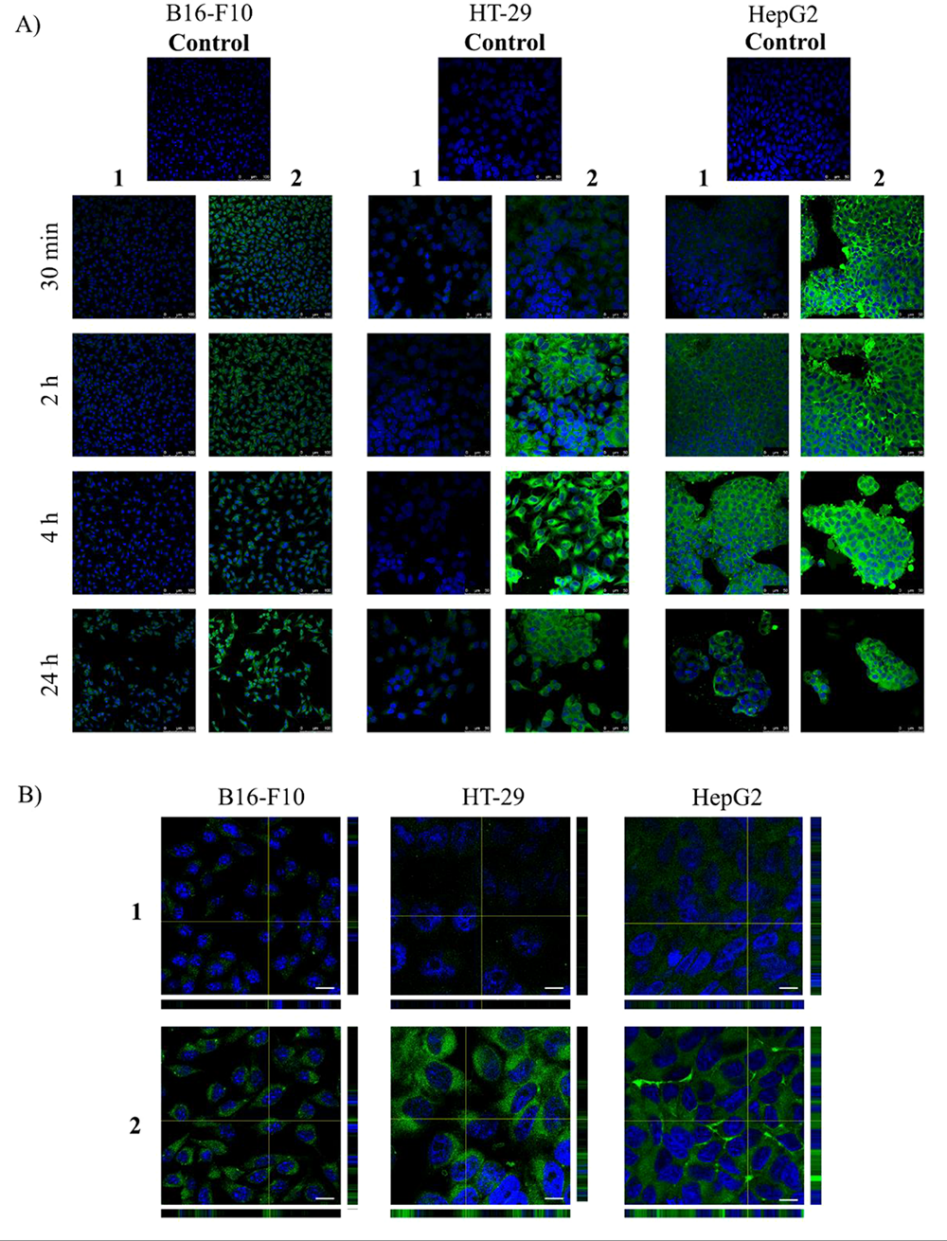
Localization of conjugates **1** and **2** in B16-F10, HT-29, and HepG2 cells. A. Confocal images of the different cell lines incubated with conjugates **1** or **2** (green) at the indicated time points. Nuclei were stained with DAPI (blue). B. High magnification of confocal images and orthogonal projections of each cell line incubated with the compounds for 2 h revealed both green fluorescent compounds inside the cell. Scale bars: A, 50 μm: B, 10 μm.

The uptake and distribution of the green fluorescence of conjugates **1** and **2** in the three cancer cell lines were analyzed by confocal microscopy at different times: 30 min and 2, 4, and 24 h (Figure 6). This temporal sequence allowed a determination of when the permeabilization of these compounds (**1** and **2**) into the cancer cells occurred. According to the cell-death studies, the maximum time chosen to perform these assays was 24 h, because after this time, apoptotic effects were detected in the cancer cells. No endogenous green fluorescence was detected in the untreated cells (Figure 6A). The effective permeabilization of the two compounds into the cells was visualized as a homogeneous green fluorescence inside the cells, according to a cytoplasmic distribution, which was verified in orthogonal projections of confocal microscopy (Figure 6B). The two compounds were visualized in the cytoplasm of the cells that remained attached in the cultures from 30 min to 24 h. However, due to the cytotoxicity of these compounds (**1** and **2**), the cell density decreased in the three cancer cell lines as the treatment time increased. Additionally, the intensity of the green fluorescence associated with conjugate **2** was higher than that of conjugate **1**, in agreement with the data from the study of the optical properties of these conjugates (Figure 3). These results were similar in the three cancer cell lines.

### Conclusions

A total of 12 NBD-Aa-triterpene conjugates of **OA** or **MA** were tested for their optical fluorescence properties and their biological activities against cell proliferation in three cancer cell lines (B16-F10, HT-29, and HepG2). The fluorescence of these derivatives (**1**–**12**) decreased as the length of the ω-amino acid chain increased. **MA** conjugate **2** was more fluorescent than **OA** conjugate **1**. The same trend was found for the rest of the **OA** and **MA** conjugates. The most promising cytotoxicity results were achieved using NBD-6AHA-triterpene conjugates **1** and **2**, with the shortest-chain ω-amino acid linker (6AHA), giving IC_50_ values from 2.4 to 19.5 μM. These were between 44- and 5-fold more effective than their corresponding precursors (****OA**** or **MA**). In most cases, these NBD conjugates (**1** and **2**) exhibited viability percentages of the nontumor cells that ranged from 75% to 90%. These two conjugates (**1** and **2**) showed apoptotic effects in the treated cells, with this effect being significant after 24 h and especially after 72 h, reaching total apoptosis rates between 31 and 55%. The NBD conjugate of **MA** (**2**) showed greater apoptotic effects than did the NBD conjugate of **OA** (**1**), in the three cancer cell lines, with apoptosis percentages ranging from 48% to 55% after 72 h. The effective permeabilization of both conjugates (**1** and **2**) into the cytoplasm of the cells was visualized by a green fluorescence, although the intensity of the fluorescence signal of conjugate **2** was higher. This green fluorescence was detectable from 30 min to 24 h, although, due to the cytotoxicity of these compounds, the cell density was accordingly decreased in the three cancer cell lines. These NBD-Aa-triterpene conjugates, in addition to being used as potential antiproliferative agents for cancer cells and having little cytotoxicity in nontumor cells, are effective fluorescent probes that will enable further subcellular studies and their localization in cells and tissues.

## Experimental Section

### General Experimental Procedures

Optical rotations were measured with a PerkinElmer 241 polarimeter at 25 °C. IR spectra were recorded on a Mattson Satellite FTIR spectrometer. NMR spectra were measured in a Varian Direct Drive spectrometer (^1^H, 400 or 500 MHz; ^13^C, 100 or 125 MHz), using CDCl_3_ as a solvent. The ^13^C NMR chemical shifts were determined with the aid of DEPT, using a flip angle of 135°. The purity of the new compounds was determined with a Waters Acquity UPLC system, coupled to a Waters Synapt G2 HRMS spectrometer with ESI. The purity of all compounds was confirmed to be ≥95%. Merck silica gel 60 aluminum sheets (ref 1.16835) were used for TLC. Merck silica gel Normasil 60 (40–63 μm, ref 27623.323) was used for flash chromatography. CH_2_Cl_2_ (Fisher, D/1852/17) served as the eluent with increasing amounts of acetone (Fisher, A/0600/17), with all solvents having analytical reagent-grade purity. The commercial 4-chloro-7-nitrobenzofurazan (NBD-Cl, ref 163260) and the different ω-amino acids were purchased from Sigma-Aldrich.

### Isolation of **OA** and **MA**

Oleanolic acid (**OA**) and maslinic acid (**MA**) were isolated from olive-mill wastes, which were extracted in a Soxhlet with hexane and EtOAc. Hexane extracts were a mixture of **OA** and **MA** (80:20), whereas this relationship was (20:80) for the EtOAc extracts. Both products were purified by column chromatography over silica gel, eluting with CH_2_Cl_2_/acetone mixtures of increasing polarity.^42^

### Synthesis of **BO** and **BM**

BnCl (418 μL) was added at a relationship of 2:1 to a solution of **OA** or **MA** (2.0 mmol) in DMF (8 mL) with K_2_CO_3_ (0.61 g). Each reaction was stirred for 4 h at 55 °C. The mixtures were diluted with water and extracted with CH_2_Cl_2_, and the respective organic layers were dried with anhydrous Na_2_SO_4_. Each solvent was removed under reduced pressure, and each residue was purified by column chromatography using CH_2_Cl_2_/acetone (10:1) to give benzyl oleanolate (**BO**, 84%)^43^ or benzyl maslinate (**BM**, 82%),^44^ as white solids.

### Synthesis of NBD-Aa-Triterpene Conjugates **1**–**12**

The different NBD-Aa-triterpene conjugates (**1**–**12**) were synthesized through a one-pot labeling protocol.^38^ A solution of NBD-Cl (1.0 mmol) in acetonitrile was added dropwise to a solution of the corresponding ω-amino acid (1.0 mmol) and sodium bicarbonate (3.0 mmol) in water. Then, the reaction mixture was incubated at 55 °C for 1 h and the acetonitrile was evaporated under reduced pressure. The aqueous reaction mixture was adjusted to approximately pH 2.0 (using 1 N HCl) and then concentrated to dryness. The crude deep orange solid was redissolved in a minimal amount of acetonitrile and dried again. Then, in the same flask, Yamaguchi coupling was performed by adding to the dry crude solid the corresponding triterpene compound (**OA**, **MA**, **BO**, or **BM**; 0.75 mmol) and dissolved and stirred in anhydrous THF, under an inert atmosphere. Then, 2,4,6-trichlorobenzoyl chloride (1.0 mmol) and anhydrous triethylamine (1.0 mmol) were successively added dropwise to the reaction mixture. After 5 min, 4-dimethylaminopyridine (1.0 mmol) was added to the reaction mixture and stirred for 12 h. The reaction was stopped by adding a few drops of water. Finally, the reaction mixture was concentrated under reduced pressure to dryness, and the different NBD-Aa-triterpene conjugates were purified on a silica gel flash column, using mixtures of CH_2_Cl_2_ and acetone of increasing polarity. The NBD-Aa-triterpene conjugates (**1**–**12**) were obtained in good yields (72%–86%).

#### 3β-((6-((7-Nitrobenzo[*c*][1,2,5]oxadiazol-4-yl)amino)hexanoyl)oxy)olean-12-en-28-oic Acid (**1**, 81%)

Orange syrup; [α]_D_^20^ +11 (*c* 1.0, CHCl_3_); IR (film) ν_max_ 3317, 2924, 1727, 1531, 1506, 1462, 1261, 1163, 1017, 736 cm^–1^; ^1^H NMR (CDCl_3_, 500 MHz) δ 8.50 and 6.18 (each 1H, AB system, *J* = 8.6 Hz, H-5″ and H-6″), 6.33 (1H, bs, NH), 5.29 (1H, dd, *J* = 3.5, 3.5 Hz, H-12), 4.51 (1H, dd, *J* = 8.0, 8.0 Hz, H-3), 3.52 (2H, c, *J* = 6.4 Hz, 2H-6′), 2.82 (1H, dd, *J* = 12.0, 4.0 Hz, H-18), 2.36 (2H, t, *J* = 7.2 Hz, 2H-2′), 1.14 (3H, s, 3H-27), 0.93, 0.91, 0.88 (each 3H, s, 3H-23, 3H-25 and 3H-29), 0.84 and 0.83 (each 3H, s, 3H-24 and 3H-30), 0.76 (3H, s, 3H-26); ^13^C NMR (CDCl_3_, 125 MHz) δ 182.4 (C, C-28), 173.3 (C, C-1′), 144.4, 144.0, and 143.9 (C, C-4a″, C-4″, and C-7a″), 143.8 (C, C-13), 136.6 (CH, C-6″), 124.3 (C, C-7″), 122.7 (CH, C-12), 98.7 (CH, C-5″), 81.2 (CH, C-3), 55.4 (CH, C-5), 47.7 (CH, C-9), 46.6 (C, C-17), 46.0 (CH2, C-19), 43.8 (CH2, C-6′), 41.8 (C, C-14), 41.2 (CH, C-18), 39.4 (C, C-8), 38.2 (CH2, C-1), 37.9 (C, C-4), 37.1 (C, C-10), 34.5 (CH2, C-2′), 33.9 (CH2, C-21), 33.2 (CH3, C-29), 32.7 (CH2, C-7), 32.6 (CH2, C-22), 30.8 (C, C-20), 28.3 (CH2, C-2), 28.2 (CH3, C-23), 27.8 and 24.5 (CH2, C-4′ and C-5′), 26.5 (CH2, C-15), 26.0 (CH3, C-27), 23.7 (CH2, C-11), 23.7 (CH3, C-30), 23.5 (CH2, C-3′), 23.1 (CH2, C-16), 18.3 (CH2, C-6), 17.2 (CH3, C-26), 16.9 (CH3, C-24), 15.5 (CH3, C-25); HRESIMS *m*/*z* 755.4351 (calcd for C_42_H_60_N_4_O_7_Na [M + Na]^+^, 755.4360).

#### 3β-Hydroxy-2α-((6-((7-nitrobenzo[*c*][1,2,5]oxadiazol-4-yl)amino)hexanoyl)oxy)olean-12-en-28-oic Acid (**2**, 76%)

Orange syrup; [α]_D_^20^ +26 (*c* 1.0, CHCl_3_); IR (film) ν_max_ 3314, 2925, 1697, 1531, 1498, 1453, 1275, 1162, 1031, 739 cm^–1^; ^1^H NMR (CDCl_3_, 500 MHz) δ 8.48 and 6.16 (each 1H, AB system, *J* = 8.6 Hz, H-5″ and H-6″), 6.69 (1H, bs, NH), 5.26 (1H, bs, H-12), 5.01 (1H, ddd, *J* = 10.0, 10.0, 4.8 Hz, H-2), 3.51 (2H, m, 2H-6′), 3.20 (1H, d, *J* = 10.0 Hz, H-3), 2.82 (1H, dd, *J* = 13.6, 4.0 Hz, H-18), 2.38 (2H, m, 2H-2′), 1.12 (3H, s, 3H-27), 1.05 and 1.04 (each 3H, s, 3H-23 and 3H-25), 0.92, 0.90, 0.88, (each 3H, s, 3H-24, 3H-29 and 3H-30), 0.75 (3H, s, 3H-26); ^13^C NMR (CDCl_3_, 125 MHz) δ 183.2 (C, C-28), 174.2 (C, C-1′), 144.4, 144.1, and 144.1 (C, C-4a″, C-4″, and C-7a″), 144.0 (C, C-13), 136.6 (CH, C-6″), 124.0 (C, C-7″), 122.3 (CH, C-12), 98.7 (CH, C-5″), 81.4 (CH, C-3), 73.2 (CH, C-2), 55.3 (CH, C-5), 47.7 (CH, C-9), 46.6 (C, C-17), 46.0 (CH2, C-19), 43.9 (CH2, C-1), 43.7 (CH2, C-6′), 41.8 (C, C-14), 41.1 (CH, C-18), 40.0 (C, C-4), 39.4 (C, C-8), 38.5 (C, C-10), 34.3 (CH2, C-2′), 33.9 (CH2, C-21), 33.2 (CH3, C-29), 32.6 (CH2, C-7), 32.5 (CH2, C-22), 30.8 (C, C-20), 28.7 (CH3, C-23), 28.0 and 26.2 (CH2, C-4′and C-5′), 27.8 (CH2, C-15), 26.1 (CH3, C-27), 24.4 (CH2, C-11), 23.7 (CH3, C-30), 23.6 (CH2, C-3′), 23.0 (CH2, C-16), 18.4 (CH2, C-6), 17.3 (CH3, C-26), 16.8 (CH3, C-24), 16.5 (CH3, C-25); HRESIMS *m*/*z* 771.4307 (calcd for C_42_H_60_N_4_O_8_Na [M + Na]^+^, 771.4309).

#### Benzyl 3β-((6-((7-Nitrobenzo[*C*][1,2,5]oxadiazol-4-yl)amino)hexanoyl)oxy)olean-12-en-28-oate (**3**, 82%)

Orange syrup; [α]_D_^20^ +22 (*c* 1.0, CHCl_3_); IR (film) ν_max_ 3349, 3066, 2925, 1723, 1581, 1455, 1255, 1158, 1029, 750 cm^–1^; ^1^H NMR (CDCl_3_, 500 MHz) δ 8.50 and 6.17 (each 1H, AB system, *J* = 8.6 Hz, H-5″ and H-6″), 7.34–7.28 (5H, m, aromatic protons of benzyl group), 6.33 (1H, m, NH), 5.28 (1H, dd, *J* = 3.6, 3.6 Hz, H-12), 5.09 and 5.04 (each 1H, AB system, *J* = 12.6 Hz, CH_2_ of benzyl group), 4.51 (1H, dd, *J* = 8.0, 8.0 Hz, H-3), 3.52 (2H, c, *J* = 6.3 Hz, 2H-6′), 2.90 (1H, dd, *J* = 14.8, 5.0 Hz, H-18), 2.35 (2H, t, *J* = 7.1 Hz, 2H-2′), 1.12 (3H, s, 3H-27), 0.92 (3H, s) and 0.89 (6H, s) (3H-23, 3H-25 and 3H-29), 0.84 (6H, s, 3H-24 and 3H-30), 0.60 (3H, s, 3H-26); ^13^C NMR (CDCl_3_, 125 MHz) δ 177.6 (C, C-28), 173.3 (C, C-1′), 144.4, 144.0, and 143.9 (C, C-4a″, C-4″, and C-7a″), 143.9 (C, C-13), 136.6 (CH, C-6″), 136.5, 128.6, 128.1, and 128.0 (aromatic carbons of benzyl group), 124.3 (C, C-7″), 122.5 (CH, C-12), 98.7 (CH, C-5″), 81.2 (CH, C-3), 66.1 (CH_2_ of benzyl group), 55.4 (CH, C-5), 47.7 (CH, C-9), 46.9 (C, C-17), 46.0 (CH2, C-19), 43.8 (CH2, C-6′), 41.8 (C, C-14), 41.5 (CH, C-18), 39.4 (C, C-8), 38.2 (CH2, C-1), 37.9 (C, C-4), 37.0 (C, C-10), 34.5 (CH2, C-2′), 34.0 (CH2, C-21), 33.2 (CH3, C-29), 32.8 (CH2, C-7), 32.5 (CH2, C-22), 30.8 (C, C-20), 28.3 (CH2, C-2), 28.2 (CH3, C-23), 27.7 and 24.5 (CH2, C-4′and C-5′), 26.5 (CH2, C-15), 26.0 (CH3, C-27), 23.8 (CH3, C-30), 23.7 (CH2, C-11), 23.5 (CH2, C-3′), 23.2 (CH2, C-16), 18.3 (CH2, C-6), 17.0 (CH3, C-26), 16.9 (CH3, C-24), 15.5 (CH3, C-25); HRESIMS *m*/*z* 845.4825 (calcd for C_49_H_66_N_4_O_7_Na [M + Na]^+^, 845.4829).

#### Benzyl 3β-Hydroxy-2α-((6-((7-nitrobenzo[*c*][1,2,5]oxadiazol-4-yl)amino)hexanoyl)oxy)olean-12-en-28-oate (**4**, 77%)

Orange syrup; [α]_D_^20^ +29 (*c* 1.0, CHCl_3_); IR (film) ν_max_ 3322, 3065, 2944, 1718, 1581, 1448, 1259, 1159, 1015, 736 cm^–1^; ^1^H NMR (CDCl_3_, 500 MHz) δ 8.48 and 6.17 (each 1H, AB system, *J* = 8.6 Hz, H-5″ and H-6″), 7.34–7.28 (5H, m, aromatic protons of benzyl group), 6.57 (1H, bs, NH), 5.27 (1H, bs, H-12), 5.09 and 5.05 (each 1H, AB system, *J* = 12.5 Hz, CH_2_ of benzyl group), 5.01 (1H, ddd, *J* = 9.8, 9.8, 4.1 Hz, H-2), 3.51 (2H, c, *J* = 6.0 Hz, 2H-6′), 3.20 (1H, d, *J* = 9.8 Hz, H-3), 2.90 (1H, dd, *J* = 13.6, 3.9 Hz, H-18), 2.38 (2H, m, 2H-2′), 1.11 (3H, s, 3H-27), 1.05 (3H, s, 3H-23), 1.01 (3H, s, 3H-25), 0.92 (3H, s, 3H-30), 0.89 (6H, s, 3H-24 and 3H-29), 0.59 (3H, s, 3H-26); ^13^C NMR (CDCl_3_, 125 MHz) δ 177.5 (C, C-28), 174.1 (C, C-1′), 144.4, 144.1, and 144.1 (C, C-4a″, C-4″, and C-7a″), 144.0 (C, C-13), 136.6 (CH, C-6″), 136.5, 128.6, and 128.2 (aromatic carbons of benzyl group), 124.2 (C, C-7″), 122.2 (CH, C-12), 98.7 (CH, C-5″), 81.4 (CH, C-3), 73.2 (CH, C-2), 66.1 (CH_2_ of benzyl group), 55.3 (CH, C-5), 47.7 (CH, C-9), 46.8 (C, C-17), 46.1 (CH2, C-19), 44.0 (CH2, C-1), 43.7 (CH2, C-6′), 41.9 (C, C-14), 41.5 (CH, C-18), 40.1 (C, C-4), 39.5 (C, C-8), 38.5 (C, C-10), 34.3 (CH2, C-2′), 34.0 (CH2, C-21), 33.3 (CH3, C-29), 32.7 (CH2, C-7), 32.5 (CH2, C-22), 30.9 (C, C-20), 28.7 (CH3, C-23), 28.0 and 26.2 (CH2, C-4′and C-5′), 27.7 (CH2, C-15), 26.0 (CH3, C-27), 24.4 (CH2, C-11), 23.8 (CH3, C-30), 23.7 (CH2, C-3′), 23.2 (CH2, C-16), 18.5 (CH2, C-6), 17.0 (CH3, C-26), 16.8 (CH3, C-24), 16.5 (CH3, C-25); HRESIMS *m*/*z* 861.4769 (calcd for C_49_H_66_N_4_O_8_Na [M + Na]^+^, 861.4778).

#### 3β-((8-((7-Nitrobenzo[*c*][1,2,5]oxadiazol-4-yl)amino)octanoyl)oxy)olean-12-en-28-oic Acid (**5**, 83%)

Orange syrup; [α]_D_^20^ +28 (*c* 1.0, CHCl_3_); IR (film) ν_max_ 3316, 2925, 1723, 1531, 1499, 1447, 1275, 1186, 1010, 739 cm^–1^; ^1^H NMR (CDCl_3_, 400 MHz) δ 8.49 and 6.17 (each 1H, AB system, *J* = 8.6 Hz, H-5″ and H-6″), 6.32 (1H, bs, NH), 5.27 (1H, dd, *J* = 3.3, 3.3 Hz, H-12), 4.50 (1H, dd, *J* = 7.9, 7.9 Hz, H-3), 3.49 (2H, c, *J* = 6.6 Hz, 2H-8′), 2.82 (1H, dd, *J* = 13.7, 3.9 Hz, H-18), 2.30 (2H, t, *J* = 7.3 Hz, 2H-2′), 1.13 (3H, s, 3H-27), 0.93 (6H, s) and 0.90 (3H, s) (3H-23, 3H-25 and 3H-29), 0.85 and 0.84 (each 3H, s, 3H-24 and 3H-30), 0.75 (3H, s, 3H-26); ^13^C NMR (CDCl_3_, 100 MHz) δ 183.1 (C, C-28), 173.6 (C, C-1′), 144.4, 144.1, and 144.0 (C, C-4a″, C-4″, and C-7a″), 143.8 (C, C-13), 136.6 (CH, C-6″), 124.2 (C, C-7″), 122.7 (CH, C-12), 98.7 (CH, C-5″), 80.9 (CH, C-3), 55.4 (CH, C-5), 47.7 (CH, C-9), 46.7 (C, C-17), 46.0 (CH2, C-19), 44.1 (CH2, C-8′), 41.8 (C, C-14), 41.2 (CH, C-18), 39.4 (C, C-8), 38.2 (CH2, C-1), 37.9 (C, C-4), 37.1 (C, C-10), 34.8 (CH2, C-2′), 33.9 (CH2, C-21), 33.2 (CH3, C-29), 32.7 and 32.6 (CH2, C-7 and C-22), 30.8 (C, C-20), 29.0 and 28.9 (CH2, C-4′and C-5′), 28.6 (CH2, C-2), 28.2 (CH3, C-23), 27.8 (CH2, C-15), 26.8 (CH2, C-7′), 26.0 (CH3, C-27), 25.0 (CH2, C-6′), 23.7 (CH3, C-30), 23.7 (CH2, C-3′), 23.5 (CH2, C-11), 23.1 (CH2, C-16), 18.3 (CH2, C-6), 17.2 (CH3, C-26), 16.9 (CH3, C-24), 15.5 (CH3, C-25); HRESIMS *m*/*z* 783.4667 (calcd for C_44_H_64_N_4_O_7_Na [M + Na]^+^, 783.4673).

#### 3β-Hydroxy-2α-((8-((7-nitrobenzo[*c*][1,2,5]oxadiazol-4-yl)amino)octanoyl)oxy)olean-12-en-28-oic Acid (**6**, 74%)

Orange syrup; [α]_D_^20^ +20 (*c* 1.0, CHCl_3_); IR (film) ν_max_ 3315, 2925, 1695, 1530, 1498, 1448, 1260, 1158, 1030, 738 cm^–1^; ^1^H NMR (CDCl_3_, 400 MHz) δ 8.49 and 6.17 (each 1H, AB system, *J* = 8.6 Hz, H-5″ and H-6″), 6.35 (1H, bs, NH), 5.26 (1H, dd, *J* = 3.7, 3.7 Hz, H-12), 4.96 (1H, ddd, *J* = 10.0, 10.0, 4.6 Hz, H-2), 3.49 (2H, c, *J* = 6.7 Hz, 2H-8′), 3.20 (1H, d, *J* = 10.0 Hz, H-3), 2.82 (1H, dd, *J* = 13.9, 4.6 Hz, H-18), 2.33 (2H, t, *J* = 7.2 Hz, 2H-2′), 1.12 (3H, s, 3H-27), 1.04 and 1.03 (each 3H, s, 3H-23 and 3H-25) 0.92 and 0.90 (each 3H, s, 3H-29 and 3H-30), 0.85 (3H, s, 3H-24), 0.74 (3H, s, 3H-26); ^13^C NMR (CDCl_3_, 100 MHz) δ 183.5 (C, C-28), 174.5 (C, C-1′), 144.4, 144.1, and 144.1 (C, C-4a″, C-4″, and C-7a″), 144.0 (C, C-13), 136.6 (CH, C-6″), 124.1 (C, C-7″), 122.3 (CH, C-12), 98.7 (CH, C-5″), 81.1 (CH, C-3), 73.2 (CH, C-2), 55.3 (CH, C-5), 47.7 (CH, C-9), 46.6 (C, C-17), 46.0 (CH2, C-19), 44.1 (CH2, C-8′), 43.8 (CH2, C-1), 41.8 (C, C-14), 41.1 (CH, C-18), 39.9 (C, C-4), 39.5 (C, C-8), 38.5 (C, C-10), 34.6 (CH2, C-2′), 33.9 (CH2, C-21), 33.2 (CH3, C-29), 32.6 (CH2, C-7 and C-22), 30.8 (C, C-20), 28.9 (CH2, C-4′and C-5′), 28.7 (CH3, C-23), 28.5 (CH2, C-7′), 27.8 (CH2, C-15), 26.8 (CH2, C-6′), 26.1 (CH3, C-27), 24.9 (CH2, C-3′), 23.7 (CH3, C-30), 23.6 (CH2, C-11), 23.0 (CH2, C-16), 18.4 (CH2, C-6), 17.3 (CH3, C-26), 16.8 (CH3, C-24), 16.5 (CH3, C-25); HRESIMS *m*/*z* 799.4611 (calcd for C_44_H_64_N_4_O_8_Na [M + Na]^+^, 799.4622).

#### Benzyl 3β-((8-((7-Nitrobenzo[*c*][1,2,5]oxadiazol-4-yl)amino)octanoyl)oxy)olean-12-en-28-oate (**7**, 85%)

Orange syrup; [α]_D_^20^ +40 (*c* 1.0, CHCl_3_); IR (film) ν_max_ 3321, 3063, 2929, 1721, 1580, 1447, 1262, 1158, 1030, 737 cm^–1^; ^1^H NMR (CDCl_3_, 500 MHz) δ 8.48 and 6.17 (each 1H, Ab system, *J* = 8.6 Hz, H-5″ and H-6″), 7.34–7.28 (5H, m, aromatic protons of benzyl group), 6.36 (1H, bs, NH), 5.27 (1H, bs, H-12), 5.09 and 5.04 (each 1H, AB system, *J* = 12.6 Hz, CH_2_ of benzyl group), 4.48 (1H, dd, *J* = 7.8, 7.8 Hz, H-3), 3.49 (2H, c, *J* = 6.4 Hz, 2H-8′), 2.89 (1H, dd, *J* = 14.3, 3.5 Hz, H-18), 2.30 (2H, t, *J* = 7.8 Hz, 2H-2′), 1.11 (3H, s, 3H-27), 0.91 (3H, s) and 0.89 (6H, s) (3H-23, 3H-25 and 3H-29), 0.84 (6H, s, 3H-24 and 3H-30), 0.60 (3H, s, 3H-26); ^13^C NMR (CDCl_3_, 125 MHz) δ 177.6 (C, C-28), 173.6 (C, C-1′), 144.4, 144.0, and 143.8 (C, C-4a″, C-4″, and C-7a″), 143.8 (C, C-13), 136.6 (CH, C-6″), 136.5, 128.5, 128.1, and 128.0 (aromatic carbons of benzyl group), 124.1 (C, C-7″), 122.5 (CH, C-12), 98.6 (CH, C-5″), 80.9 (CH, C-3), 66.0 (CH_2_ of benzyl group), 55.4 (CH, C-5), 47.6 (CH, C-9), 46.9 (C, C-17), 46.0 (CH2, C-19), 44.1 (CH2, C-8′), 41.8 (C, C-14), 41.5 (CH, C-18), 39.4 (C, C-8), 38.2 (CH2, C-1), 37.8 (C, C-4), 37.0 (C, C-10), 34.8 (CH2, C-2′), 34.0 (CH2, C-21), 33.2 (CH3, C-29), 32.8 and 32.5 (CH2, C-7 and C-22), 30.8 (C, C-20), 29.0 and 28.9 (CH2, C-4′and C-5′), 28.5 (CH2, C-2), 28.2 (CH3, C-23), 27.7 (CH2, C-15), 26.8 and 25.0 (CH2, C-6′and C-7′), 26.0 (CH3, C-27), 23.8 (CH3, C-30), 23.7 (CH2, C-3′), 23.5 (CH2, C-11), 23.2 (CH2, C-16), 18.3 (CH2, C-6), 17.0 (CH3, C-26), 16.9 (CH3, C-24), 15.5 (CH3, C-25); HRESIMS *m*/*z* 873.5142 (calcd for C_51_H_70_N_4_O_7_Na [M + Na]^+^, 873.5142).

#### Benzyl 3β-Hydroxy-2α-((8-((7-nitrobenzo[*c*][1,2,5]oxadiazol-4-yl)amino)octanoyl)oxy)olean-12-en-28-oate (**8**, 81%)

Orange syrup; [α]_D_^20^ +11 (*c* 1.0, CHCl_3_); IR (film) ν_max_ 3321, 3063, 2929, 1717, 1581, 1448, 1261, 1157, 1030, 735 cm^–1^; ^1^H NMR (CDCl_3_, 500 MHz) δ 8.50 and 6.17 (each 1H, AB system, *J* = 8.6 Hz, H-5″ and H-6″), 7.34–7.28 (5H, m, aromatic protons of benzyl group), 6.34 (1H, bs, NH), 5.26 (1H, bs, H-12), 5.09 and 5.04 (each 1H, AB system, *J* = 12.5 Hz, CH_2_ of benzyl group), 4.96 (1H, ddd, *J* = 10.0, 10.0, 4.3 Hz, H-2), 3.51 (2H, c, *J* = 6.3 Hz, 2H-8′), 3.18 (1H, d, *J* = 10.0 Hz, H-3), 2.90 (1H, dd, *J* = 13.8, 3.9 Hz, H-18), 2.33 (2H, t, *J* = 7.3 Hz, 2H-2′), 1.11 (3H, s, 3H-27), 1.04 (3H, s, 3H-23), 0.99 (3H, s, 3H-25), 0.91 (3H, s, 3H-30), 0.89 (3H, s, 3H-29), 0.85 (3H, s, 3H-24), 0.58 (3H, s, 3H-26); ^13^C NMR (CDCl_3_, 125 MHz) δ 177.5 (C, C-28), 174.5 (C, C-1′), 144.4, 144.1, and 144.1 (C, C-4a″, C-4″, and C-7a″), 144.0 (C, C-13), 136.6 (CH, C-6″), 136.5, 128.5, 128.2, and 128.1 (aromatic carbons of benzyl group), 124.2 (C, C-7″), 122.2 (CH, C-12), 98.6 (CH, C-5″), 81.1 (CH, C-3), 73.2 (CH, C-2), 66.1 (CH_2_ of benzyl group), 55.3 (CH, C-5), 47.7 (CH, C-9), 46.8 (C, C-17), 46.0 (CH2, C-19), 44.0 (CH2, C-8′), 43.9 (CH2, C-1), 41.9 (C, C-14), 41.5 (CH, C-18), 39.9 (C, C-4), 39.5 (C, C-8), 38.4 (C, C-10), 34.6 (CH2, C-2′), 34.0 (CH2, C-21), 33.2 (CH3, C-29), 32.7 (CH2, C-7), 32.5 (CH2, C-22), 30.8 (C, C-20), 28.9, 28.9, 28.5, and 26.8 (CH2, C-4′/C-7′), 28.7 (CH3, C-23), 27.7 (CH2, C-15), 26.0 (CH3, C-27), 24.9 (CH2, C-3′), 23.8 (CH3, C-30), 23.6 (CH2, C-11), 23.1 (CH2, C-16), 18.4 (CH2, C-6), 17.0 (CH3, C-26), 16.8 (CH3, C-24), 16.5 (CH3, C-25); HRESIMS *m*/*z* 889.5081 (calcd for C_51_H_70_N_4_O_8_Na [M + Na]^+^, 889.5091).

#### 3β-((11-((7-Nitrobenzo[*c*][1,2,5]oxadiazol-4-yl)amino)undecanoyl)oxy)olean-12-en-28-oic Acid (**9**, 83%)

Orange syrup; [α]_D_^20^ +40 (*c* 1.0, CHCl_3_); IR (film) ν_max_ 3319, 2926, 1727, 1581, 1506, 1447, 1275, 1147, 1034, 749 cm^–1^; ^1^H NMR (CDCl_3_, 500 MHz) δ 8.48 and 6.17 (each 1H, AB system, *J* = 8.6 Hz, H-5″ and H-6″), 6.39 (1H, bs, NH), 5.26 (1H, bs, H-12), 4.49 (1H, dd, *J* = 7.8, 7.8 Hz, H-3), 3.48 (2H, c, *J* = 6.2 Hz, 2H-11′), 2.81 (1H, dd, *J* = 13.4, 3.3 Hz, H-18), 2.29 (2H, t, *J* = 7.3 Hz, 2H-2′), 1.12 (3H, s, 3H-27), 0.93, 0.92, and 0.90 (each 3H, s, 3H-23, 3H-25 and 3H-29), 0.85 and 0.84 (each 3H, s, 3H-24 and 3H-30), 0.74 (3H, s, 3H-26); ^13^C NMR (CDCl_3_, 125 MHz) δ 184.0 (C, C-28), 173.8 (C, C-1′), 144.4, 144.0, and 144.0 (C, C-4a″, C-4″, and C-7a″), 143.7 (C, C-13), 136.6 (CH, C-6″), 124.0 (C, C-7″), 122.6 (CH, C-12), 98.6 (CH, C-5″), 80.8 (CH, C-3), 55.4 (CH, C-5), 47.7 (CH, C-9), 46.7 (C, C-17), 46.0 (CH2, C-19), 44.2 (CH2, C-11′), 41.7 (C, C-14), 41.1 (CH, C-18), 39.4 (C, C-8), 38.2 (CH2, C-1), 37.9 (C, C-4), 37.1 (C, C-10), 34.9 (CH2, C-2′), 33.9 (CH2, C-21), 33.2 (CH3, C-29), 32.7 and 32.6 (CH2, C-7 and C-22), 30.8 (C, C-20), 29.5, 29.4, 29.3, 29.3, and 29.2 (CH2, C-4′/C-8′), 28.6 (CH2, C-2), 28.2 (CH3, C-23), 27.8 (CH2, C-15), 27.0 (CH2, C-10′), 26.0 (CH3, C-27), 25.2 (CH2, C-9′), 23.7 (CH2, C-3′), 23.7 (CH3, C-30), 23.5 (CH2, C-11), 23.0 (CH2, C-16), 18.3 (CH2, C-6), 17.3 (CH3, C-26), 16.9 (CH3, C-24), 15.5 (CH3, C-25); HRESIMS *m*/*z* 825.5134 (calcd for C_47_H_70_N_4_O_7_Na [M + Na]^+^, 825.5142).

#### 3β-Hydroxy-2α-((11-((7-nitrobenzo[*c*][1,2,5]oxadiazol-4-yl)amino)undecanoyl)oxy)olean-12-en-28-oic Acid (**10**, 72%)

orange syrup; [α]_D_^20^ +14 (*c* 1.0, CHCl_3_); IR (film) ν_max_ 3329, 2927, 1725, 1582, 1500, 1447, 1274, 1152, 1014, 735 cm^–1^; ^1^H NMR (CDCl_3_, 500 MHz) δ 8.50 and 6.18 (each 1H, AB system, *J* = 8.6 Hz, H-5″ and H-6″), 6.31 (1H, bs, NH), 5.30 (1H, bs, H-12), 4.95 (1H, ddd, *J* = 10.0, 10.0, 4.1 Hz, H-2), 3.49 (2H, c, *J* = 6.1 Hz, 2H-11′), 3.19 (1H, d, *J* = 10.0 Hz, H-3), 2.82 (1H, bd, *J* = 13.1 Hz, H-18), 2.31 (2H, dt, *J* = 6.6, 1.4 Hz, 2H-2′), 1.13 (3H, s, 3H-27), 1.05 (3H, s, 3H-23), 1.02 (3H, s, 3H-25), 0.92 and 0.91 (each 3H, s, 3H-29 and 3H-30), 0.86 (3H, s, 3H-24), 0.78 (3H, s, 3H-26); ^13^C NMR (CDCl_3_, 125 MHz) δ 174.6 (C, C-28), 173.0 (C, C-1′), 144.4, 144.1, and 144.0 (C, C-4a″, C-4″, and C-7a″), 143.5 (C, C-13), 136.6 (CH, C-6″), 124.1 (C, C-7″), 122.8 (CH, C-12), 98.6 (CH, C-5″), 81.1 (CH, C-3), 73.2 (CH, C-2), 55.3 (CH, C-5), 48.5 (C, C-17), 47.7 (CH, C-9), 45.9 (CH2, C-19), 44.1 (CH2, C-11′), 43.8 (CH2, C-1), 42.0 (C, C-14), 41.4 (CH, C-18), 39.9 (C, C-4), 39.6 (C, C-8), 38.5 (C, C-10), 34.7 (CH2, C-2′), 33.7 (CH2, C-21), 33.1 (CH3, C-29), 32.7 (CH2, C-7), 31.5 (CH2, C-22), 30.8 (C, C-20), 29.4, 29.3, 29.3, 29.3, and 29.2 (CH2, C-4′/C-8′), 28.7 (CH3, C-23), 28,7 (CH2, C-10′), 27.5 (CH2, C-15), 27.0 (CH2, C-9′), 26.0 (CH3, C-27), 25.1 (CH2, C-3′), 23.8 (CH3, C-30), 23.7 (CH2, C-11), 23.1 (CH2, C-16), 18.4 (CH2, C-6), 17.3 (CH3, C-26), 16.8 (CH3, C-24), 16.5 (CH3, C-25); HRESIMS *m*/*z* 841.5066 (calcd for C_47_H_70_N_4_O_8_Na [M + Na]^+^, 841.5091).

#### Benzyl 3β-((6-((7-Nitrobenzo[*c*][1,2,5]oxadiazol-4-yl)amino)undecanoyl)oxy)olean-12-en-28-oate (**11**, 86%)

Orange syrup; [α]_D_^20^ +34 (*c* 1.0, CHCl_3_); IR (film) ν_max_ 3322, 2927, 1721, 1580, 1447, 1275, 1158, 1011, 738 cm^–1^; ^1^H NMR (CDCl_3_, 500 MHz) δ 8.49 and 6.17 (each 1H, AB system, *J* = 8.7 Hz, H-5″ and H-6″), 7.34–7.28 (5H, m, aromatic protons of benzyl group), 6.26 (1H, bs, NH), 5.28 (1H, dd, *J* = 3.7, 3.7 Hz, H-12), 5.09 and 5.04 (each 1H, AB system, *J* = 12.5 Hz, CH_2_ of benzyl group), 4.49 (1H, dd, *J* = 8.0, 8.0 Hz, H-3), 3.49 (2H, c, *J* = 6.7 Hz, 2H-11′), 2.90 (1H, dd, *J* = 13.9, 4.5 Hz, H-18), 2.29 (2H, dt, *J* = 7.3, 1.1 Hz, 2H-2′), 1.12 (3H, s, 3H-27), 0.91, 0.90, and 0.89 (each 3H, s, 3H-23, 3H-25 and 3H-29), 0.85 and 0.84 (each 3H, s, 3H-24 and 3H-30), 0.61 (3H, s, 3H-26); ^13^C NMR (CDCl_3_, 125 MHz) δ 177.6 (C, C-28), 173.8 (C, C-1′), 144.4, 144.0, and 144.0 (C, C-4a″, C-4″, and C-7a″), 143.9 (C, C-13), 136.6 (CH, C-6″), 128.5, 128.1, and 128.0 (aromatic carbons of benzyl group), 124.2 (C, C-7″), 122.5 (CH, C-12), 98.6 (CH, C-5″), 80.8 (CH, C-3), 66.1 (CH_2_ of benzyl group), 55.4 (CH, C-5), 47.7 (CH, C-9), 46.9 (C, C-17), 46.0 (CH2, C-19), 44.1 (CH2, C-11′), 41.8 (C, C-14), 41.5 (CH, C-18), 39.5 (C, C-8), 38.3 (CH2, C-1), 37.9 (C, C-4), 37.1 (C, C-10), 34.9 (CH2, C-2′), 34.0 (CH2, C-21), 33.2 (CH3, C-29), 32.8 (CH2, C-7), 32.5 (CH2, C-22), 30.8 (C, C-20), 29.5, 29.4, 29.3, 29.3, and 29.2 (CH2, C-4′/C-8′), 28.7 (CH2, C-2), 28.2 (CH3, C-23), 27.8 (CH2, C-15), 27.1 (CH2, C-10′), 26.0 (CH3, C-27), 25.2 (CH2, C-9′), 23.8 (CH2, C-3′), 23.7 (CH3, C-30), 23.5 (CH2, C-11), 23.2 (CH2, C-16), 18.4 (CH2, C-6), 17.0 (CH3, C-26), 16.9 (CH3, C-24), 15.5 (CH3, C-25); HRESIMS *m*/*z* 915.5605 (calcd for C_54_H_76_N_4_O_7_Na [M + Na]^+^, 915.5612).

#### Benzyl 3β-Hydroxy-2α-((6-((7-nitrobenzo[*c*][1,2,5]oxadiazol-4-yl)amino)undecanoyl)oxy)olean-12-en-28-oate (**12**, 84%)

Orange syrup; [α]_D_^20^ +13 (*c* 1.0, CHCl_3_); IR (film) ν_max_ 3321, 2927, 1717, 1580, 1448, 1275, 1158, 1014, 738 cm^–1^; ^1^H NMR (CDCl_3_, 500 MHz) δ 8.50 and 6.17 (each 1H, AB system, *J* = 8.6 Hz, H-5″ and H-6″), 7.34–7.28 (5H, m, aromatic protons of benzyl group), 6.30 (1H, bs, NH), 5.26 (1H, dd, *J* = 3.7, 3.7 Hz, H-12), 5.09 and 5.04 (each 1H, AB system, *J* = 12.5 Hz, CH_2_ of benzyl group), 4.94 (1H, ddd, *J* = 9.9, 9.9, 4.4 Hz, H-2), 3.47 (2H, m, 2H-11′), 3.19 (1H, d, *J* = 9.9 Hz, H-3), 2.90 (1H, dd, *J* = 13.9, 4.7 Hz, H-18), 2.30 (2H, dt, *J* = 7.5, 1.7 Hz, 2H-2′), 1.11 (3H, s, 3H-27), 1.04 (3H, s, 3H-23), 0.99 (3H, s, 3H-25), 0.91 (3H, s, 3H-30), 0.89 (3H, s, 3H-29), 0.85 (3H, s, 3H-24), 0.58 (3H, s, 3H-26); ^13^C NMR (CDCl_3_, 125 MHz) δ 177.5 (C, C-28), 174.6 (C, C-1′), 144.4, 144.0, and 144.0 (C, C-4a″, C-4″, and C-7a″), 143.9 (C, C-13), 136.6 (CH, C-6″), 136.5, 128.5, 128.1, and 128.0 (aromatic carbons of benzyl group), 122.2 (CH, C-12), 98.6 (CH, C-5″), 81.0 (CH, C-3), 73.2 (CH, C-2), 66.1 (CH_2_ of benzyl group), 55.3 (CH, C-5), 47.7 (CH, C-9), 46.8 (C, C-17), 46.0 (CH2, C-19), 44.1 (CH2, C-11′), 43.8 (CH2, C-1), 41.9 (C, C-14), 41.5 (CH, C-18), 39.9 (C, C-4), 39.5 (C, C-8), 38.4 (C, C-10), 34.7 (CH2, C-2′), 34.0 (CH2, C-21), 33.2 (CH3, C-29), 32.7 (CH2, C-7), 32.5 (CH2, C-22), 30.8 (C, C-20), 29.4, 29.3, 29.3, 29.3, and 29.1 (CH2, C-4′/C-8′), 28.7 (CH3, C-23), 28.6 (CH2, C-10′), 27.7 (CH2, C-15), 27.0 (CH2, C-9′), 26.0 (CH3, C-27), 25.1 (CH2, C-3′), 23.8 (CH3, C-30), 23.6 (CH2, C-11), 23.1 (CH2, C-16), 18.4 (CH2, C-6), 17.0 (CH3, C-26), 16.8 (CH3, C-24), 16.5 (CH3, C-25); HRESIMS *m*/*z* 931.5558 (calcd for C_54_H_76_N_4_O_8_Na [M + Na]^+^, 931.5561).

### Fluorescence Spectroscopy

The UV–visible spectra were recorded on a BioSpectronic Kinetic spectrophotometer (Eppendorf, Germany). All steady-state fluorescence measurements were performed using a FluoroMax-4 spectrofluorometer (Horiba, Jobin Yvon), in the “S” mode. This apparatus was equipped with a 150-W xenon lamp and a Peltier drive to control the temperature in the cell housing. The fluorescence spectra of the compounds were recorded after excitation at 480 nm, corresponding to the wavelength of the maximum absorption.

Fluorescence lifetime of the samples was determined from time-resolved fluorescence measurements. For these, the LifeSpec II luminescence spectrometer (Edinburgh Instruments, Ltd.), equipped with a 485 nm pulsed light-emitting diode and a 100 ns pulse period, was used, recording the emission at λ_max_. The data analysis was analyzed using Edinburgh Instruments’ FAST software package. The instrumental response function (IRF) was regularly determined by measuring the scattering of a Ludox solution. All intensity-decay curves were fitted as a sum of exponential terms:
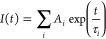
where *A*_*i*_ is a pre-exponential factor of component *i* with a lifetime τ_*i*_. In all cases, a single-exponential decay function was required for the best fit, where the reduced χ^2^ value ≤1.05 and a random distribution of weighted residuals determined the quality of the fits.

The fluorescence quantum yields (Φ_F_) of compounds were determined by the comparison method, using Coumarin 6 as a standard sample in ethanol solution (Φ_F_ = 0.78), applying the following equation:^45^
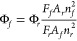
where *F* is the area under the corrected emission spectrum, *A* is the absorbance at the excitation wavelength, and *n* is the refractive index of the solvent used. The subscripts r and f refer to the reference and sample, respectively. All the photophysical experiments were performed at 25 °C and the sample solutions were placed in a quartz cuvette with a 1 cm path-length. DMSO was used as the solvent.

### Test Compounds

The different compounds used in the cell treatments were dissolved before their use in DMSO at a concentration of 5 mg/mL, constituting the stock solutions, stored at −20 °C. Before each experiment, these solutions were diluted in cell-culture medium to the appropriate concentrations for each cell assay.

### Cell Cultures

Three cancer cell lines were used to perform the tests: B16-F10 mouse melanoma cells (ATCC CRL-6475), the HT-29 human colon adenocarcinoma tumor line (ECACC 91072201), and the HepG2 human hepatocarcinoma tumor line (ECACC 85011430). All were cultured in DMEM medium supplemented with 10% heat-inactivated fetal bovine serum and gentamicin, and incubated at 37 °C in an atmosphere of 5% CO_2_ with 95% humidity.

### Cell-Viability Assay

The effect of the synthesized compounds on the viability of tumor cells was assessed using the MTT assay, based on the ability of living cells to metabolically reduce 3-(4,5-dimethylthiazole)-2,5-diphenyltetrazole bromide, rendering formazan, a colored compound with a maximum absorbance of 570 nm. To study the cytotoxic effects of the compounds in the three cancer cell lines, the different lines were seeded in 96-well plates at a cell density per well of 5 × 10^3^ for B16-F10, 6 × 10^3^ for HT-29, and 15 × 10^3^ for HepG2. After seeding, the plates were grown for 24 h and subsequently treated in triplicate with different compounds at different concentrations (0–200 μg/mL) for 72 h. After this time, the cells were stained by adding 100 mL of MTT (0.5 mg/mL) per well and incubated for 1 h. Subsequently, the cells were washed with phosphate-buffered saline (PBS), and formazan was resuspended in 100 μL of DMSO per well. Cell viability was measured by absorbance at 550 nm in an ELISA plate reader (Tecan Sunrise MR20–301, TECAN, Austria).

### Annexin V-FITC/Propidium Iodide Flow-Cytometry Analysis

Cell death (apoptosis) was studied by flow cytometry using a FACScan (fluorescence-activated cell sorter) flow cytometer (Coulter Corporation, Hialeah, FL). For this assay, 24-well plates were used, seeding at a cell density of 5 × 10^4^ for B16-F10, 6 × 10^4^ for HT-29, and 15 × 10^4^ for HepG2. The cells were incubated for 24 h with 1.5 mL of culture medium. Subsequently, the cells were treated with compounds **1** and **2** in triplicate for 4, 24, and 72 h, at the concentration of their corresponding IC_50_. The cells were collected and resuspended in a binding buffer (10 mM HEPES/NaOH, pH 7.4, 140 mM NaCl, 2.5 mM CaCl_2_). Annexin V-FITC conjugate (1 μg/mL) was then added and incubated for 1 h at room temperature in darkness. Just before analysis by flow cytometry, cells were stained with 5 μL of 1 mg/mL PI solution. In each experiment, approximately 10 × 10^3^ cells were analyzed, and the experiment was duplicated twice.

### Confocal Microscopy

Cells were seeded on coverslips in 24-well plates at a cell density per well of 5 × 10^4^ for B16-F10, 6 × 10^4^ for HT-29, and 15 × 10^4^ for HepG2. Then, cells were incubated with the fluorescent compounds for 30 min and 2, 4, and 24 h, at their IC_50_ concentrations, to analyze the cellular uptake. Cells were washed thoroughly and fixed with 4% paraformaldehyde in PBS at different type points. For the visualization of nuclei, 4′,6-diamidino-2-phenylindole (DAPI, 3.0 μM) was used as DNA-specific dye. Before the samples were visualized by confocal microscopy, 2 μL of mounting medium (mowiol) was added. Images were captured using a Leica TCS SPS confocal microscope (excitation 480 nm, emission 546 nm) with 50× magnification and then analyzed by ImageJ software (version 1.50i, NIH) to provide orthogonal projections to verify whether the compounds had penetrated the cells.

### Statistical Analysis

The results were subjected to statistical analysis and nonlinear regression to determine the concentration at which the different compounds reduced the cell population by half (IC_50_). For this, Sigmaplot 12.5 software was used, and all quantitative data were expressed as means ± standard deviation (SD). All data shown are representative of at least two independent experiments performed in triplicate.

## Supporting Information Available

^1^H NMR and ^13^C NMR spectra of compounds **1**–**12**, graphs of cell viability percentages of compounds **1**–**12** (PDF)
