# Supplementary material for: Synthesis, Optical Properties, and Antiproliferative Evaluation of NBD-Triterpene Fluorescent Probes
Source: J Nat Prod. 2022 Dec 21;86(1):166–75. doi: 10.1021/acs.jnatprod.2c00880 (PMC9887599; doi:10.1021/acs.jnatprod.2c00880)

Supporting Information

Synthesis, Optical Properties, and Antiproliferative Evaluation of NBD-Triterpene Fluorescent Probes

dedicated to Prof. Andrés García-Granados on the occasion of his retirement

Marta Medina-O'Donnell,[†] *Karina Vega-Granados,*[†] *Antonio Martinez,*[†] *M. Rosario Sepúlveda,*[‡] *José Antonio Molina-Bolívar,*[§] *Luis Álvarez de Cienfuegos,*[†] *Andres Parra,*[†] *Fernando J. Reyes-Zurita,*[*,^] *and Francisco Rivas*[*,†]

[†] Departamento de Química Orgánica. [‡] Departamento de Biología Celular. [^] Departamento de Bioquímica y Biología Molecular I. Facultad de Ciencias, Universidad de Granada, E-18071 Granada, Spain

[§] Departamento de Física Aplicada II. Escuela de Ingeniería, Universidad de Málaga, E-29071 Málaga, Spain

Table of Contents

Page S3: **Figures S1 and S2**: ^{1}H and ^{13}C NMR spectra of compound **1**.
Page S4: **Figures S3 and S4**: ^{1}H and ^{13}C NMR spectra of compound **2**.
Page S5: **Figures S5 and S6**: ^{1}H and ^{13}C NMR spectra of compound **3**.
Page S6: **Figures S7 and S8**: ^{1}H and ^{13}C NMR spectra of compound **4**.
Page S7: **Figures S9 and S10**: ^{1}H and ^{13}C NMR spectra of compound **5**.
Page S8: **Figures S11 and S12**: ^{1}H and ^{13}C NMR spectra of compound **6**.
Page S9: **Figures S13 and S14**: ^{1}H and ^{13}C NMR spectra of compound **7**.
Page S10: **Figures S15 and S16**: ^{1}H and ^{13}C NMR spectra of compound **8**.
Page S11: **Figures S17 and S18**: ^{1}H and ^{13}C NMR spectra of compound **9**.
Page S12: **Figures S19 and S20**: ^{1}H and ^{13}C NMR spectra of compound **10**.
Page S13: **Figures S21 and S22**: ^{1}H and ^{13}C NMR spectra of compound **11**.
Page S14: **Figures S23 and S24**: ^{1}H and ^{13}C NMR spectra of compound **12**.

Page S15: **Figure S25**: Graphs of cell viability percentages of compound **1** (IC_{50}) in 3 cancer-cell lines.
Page S16: **Figure S26**: Graphs of cell viability percentages of compound **2** (IC_{50}) in 3 cancer-cell lines.
Page S17: **Figure S27**: Graphs of cell viability percentages of compound **3** (IC_{50}) in 3 cancer-cell lines.
Page S18: **Figure S28**: Graphs of cell viability percentages of compound **4** (IC_{50}) in 3 cancer-cell lines.
Page S19: **Figure S29**: Graphs of cell viability percentages of compound **5** (IC_{50}) in 3 cancer-cell lines.
Page S20: **Figure S30**: Graphs of cell viability percentages of compound **6** (IC_{50}) in 3 cancer-cell lines.
Page S21: **Figure S31**: Graphs of cell viability percentages of compound **7** (IC_{50}) in 3 cancer-cell lines.
Page S22: **Figure S32**: Graphs of cell viability percentages of compound **8** (IC_{50}) in 3 cancer-cell lines.
Page S23: **Figure S33**: Graphs of cell viability percentages of compound **9** (IC_{50}) in 3 cancer-cell lines.
Page S24: **Figure S34**: Graphs of cell viability percentages of compound **10** (IC_{50}) in 3 cancer-cell lines.
Page S25: **Figure S35**: Graphs of cell viability percentages of compound **11** (IC_{50}) in 3 cancer-cell lines.
Page S26: **Figure S36**: Graphs of cell viability percentages of compound **12** (IC_{50}) in 3 cancer-cell lines.

Figure S1: ^{1}H NMR spectrum of compound **1** ($CDCl_3$, 500 MHz).

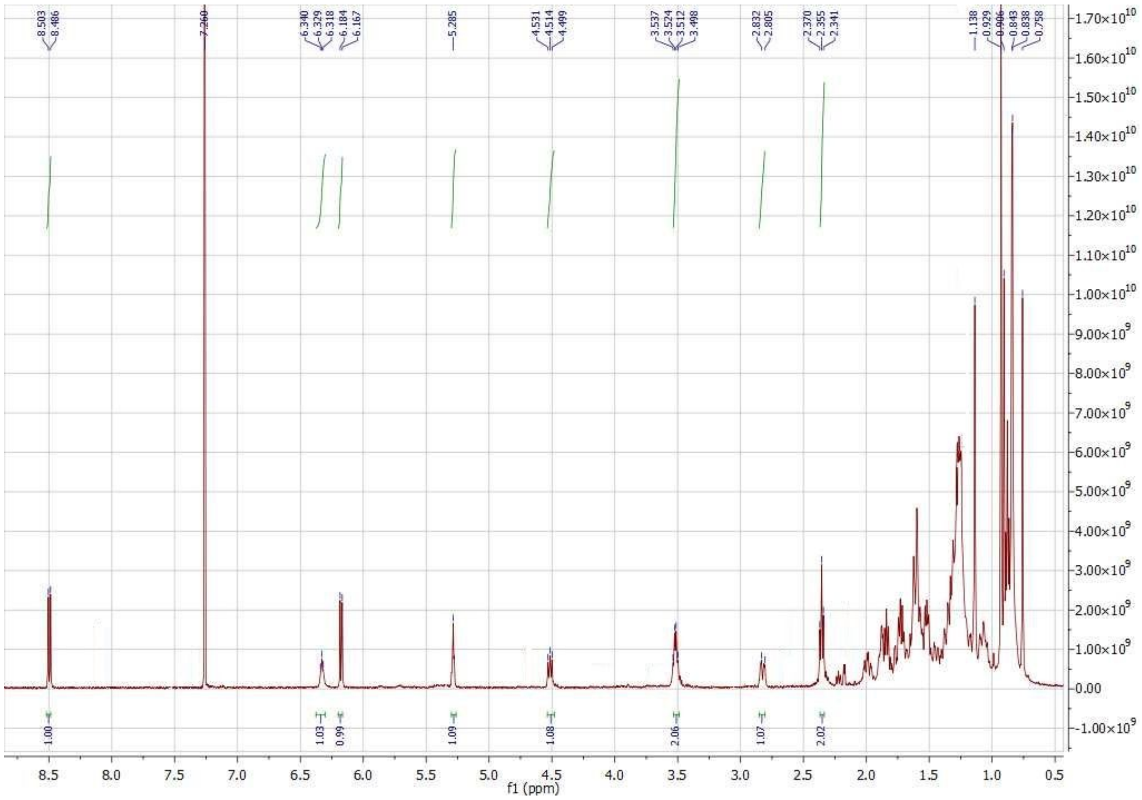

Figure S2: ^{13}C NMR spectrum of compound **1** ($CDCl_3$, 125 MHz).

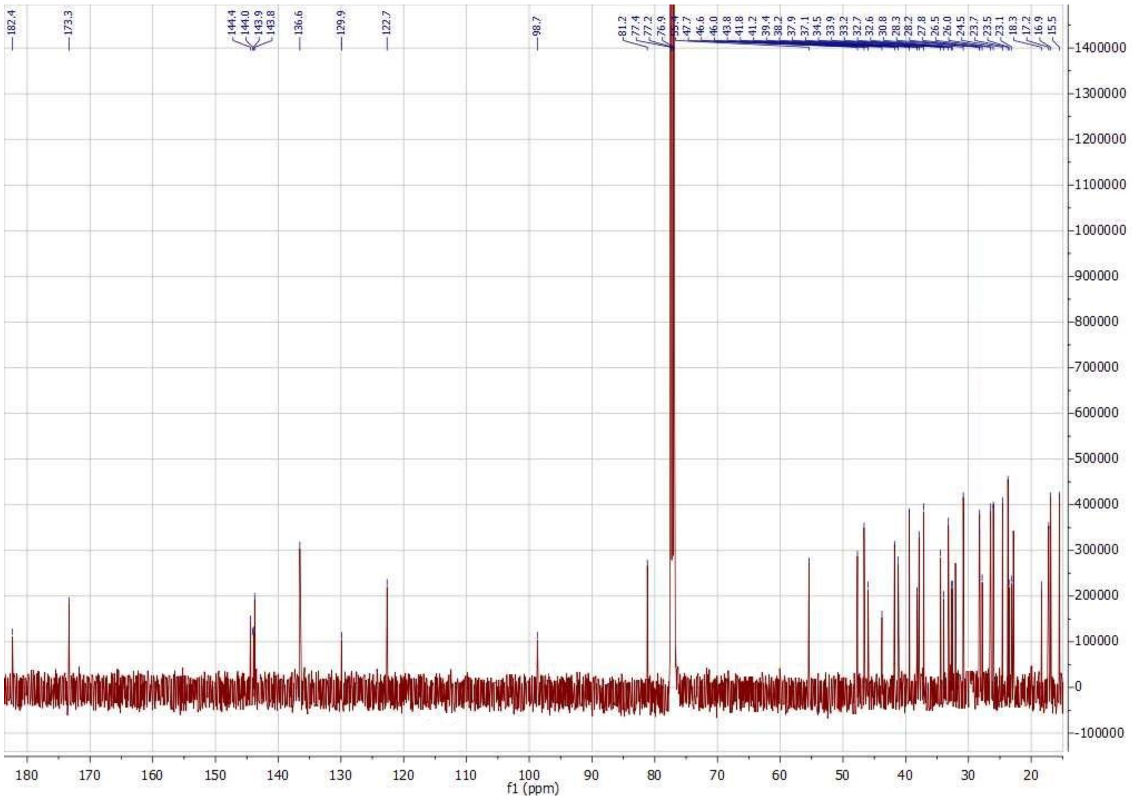

Figure S3: ^{1}H NMR spectrum of compound **2** ($CDCl_3$, 500 MHz).

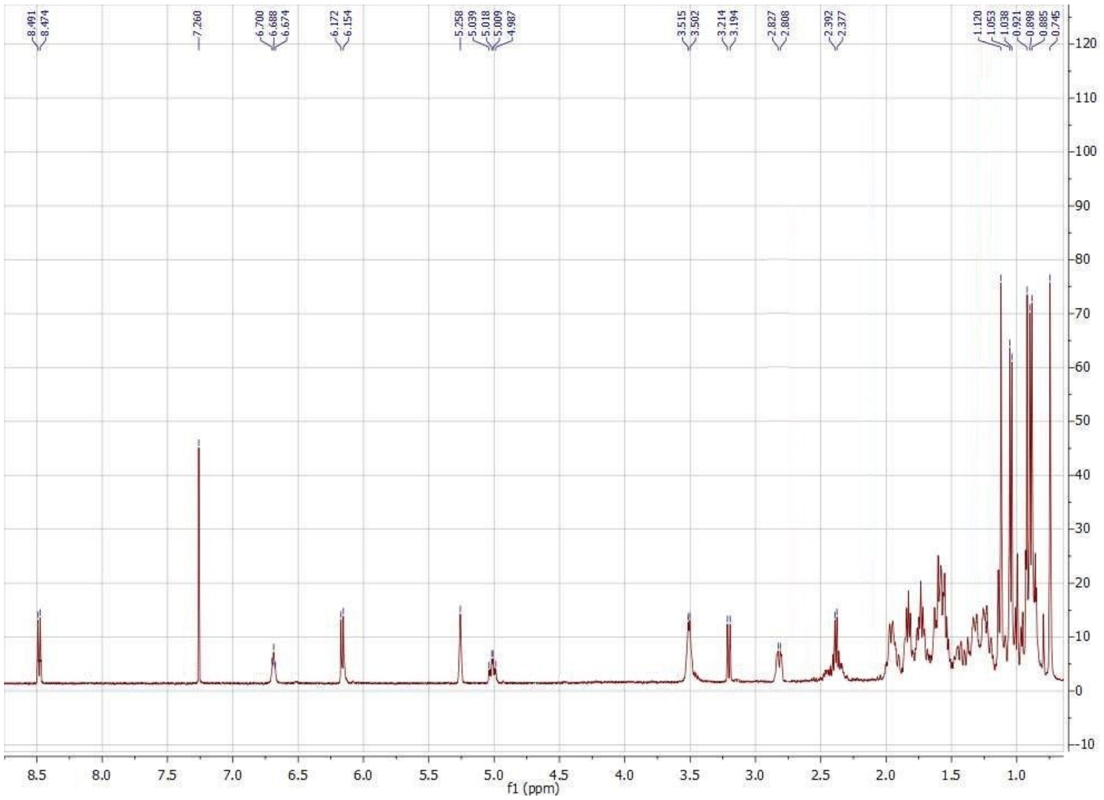

Figure S4: ^{13}C NMR spectrum of compound **2** ($CDCl_3$, 125 MHz).

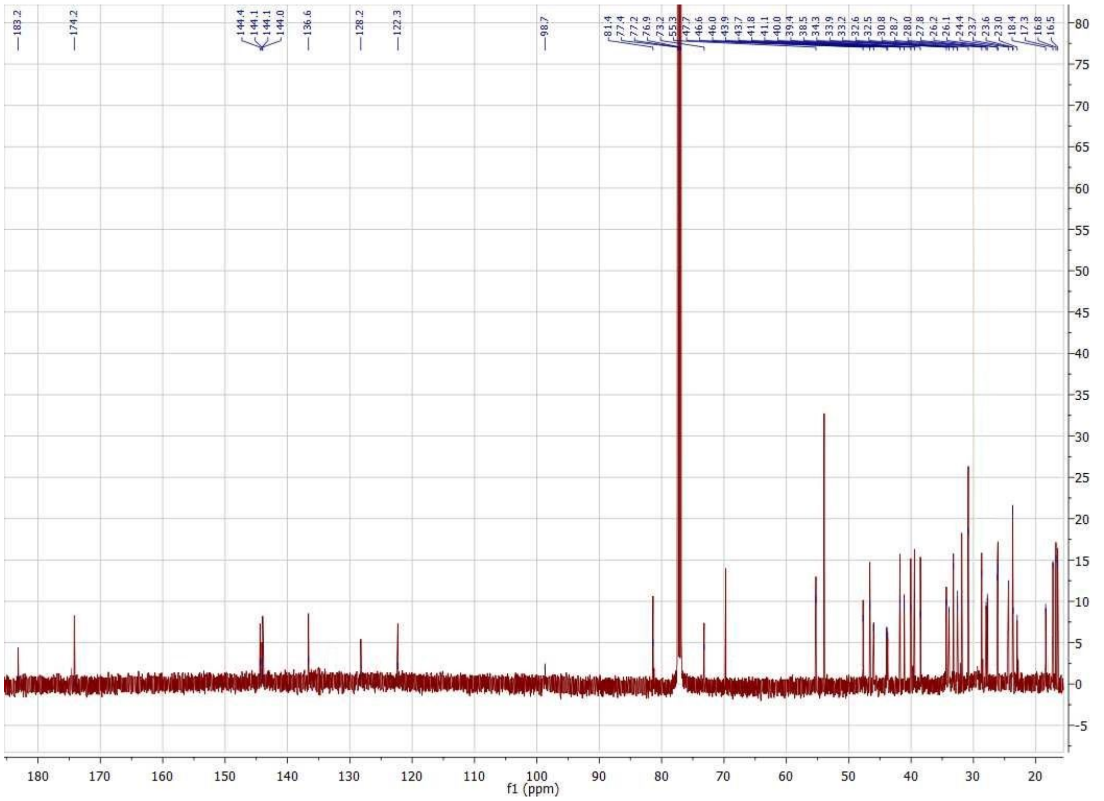

Figure S5: ^{1}H NMR spectrum of compound **3** ($CDCl_3$, 500 MHz).

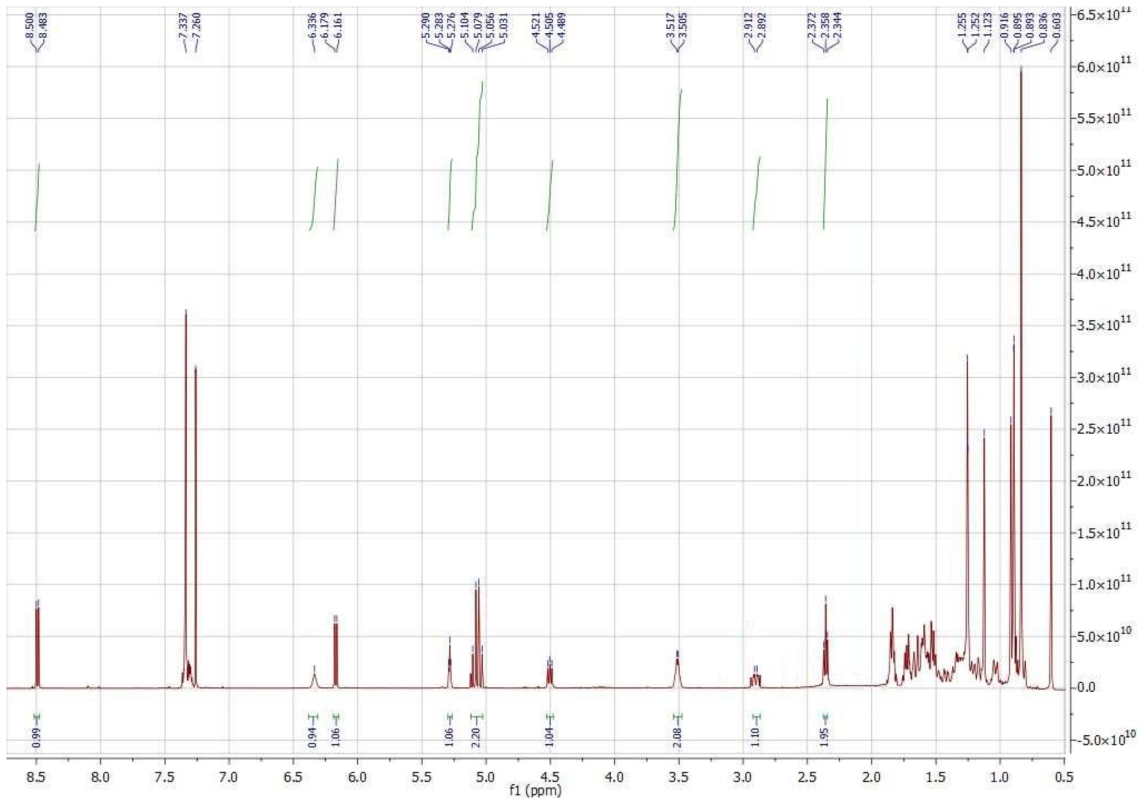

Figure S6: ^{13}C NMR spectrum of compound **3** ($CDCl_3$, 125 MHz).

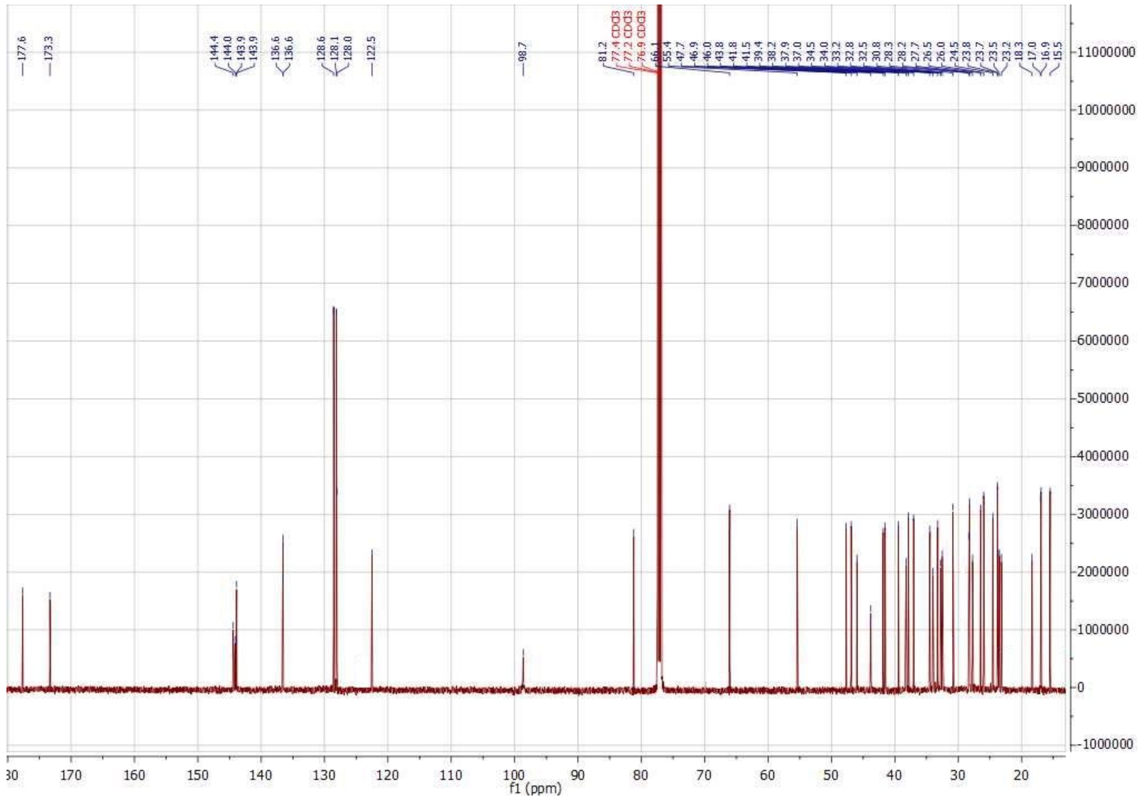

Figure S7: ^{1}H NMR spectrum of compound **4** ($CDCl_3$, 500 MHz).

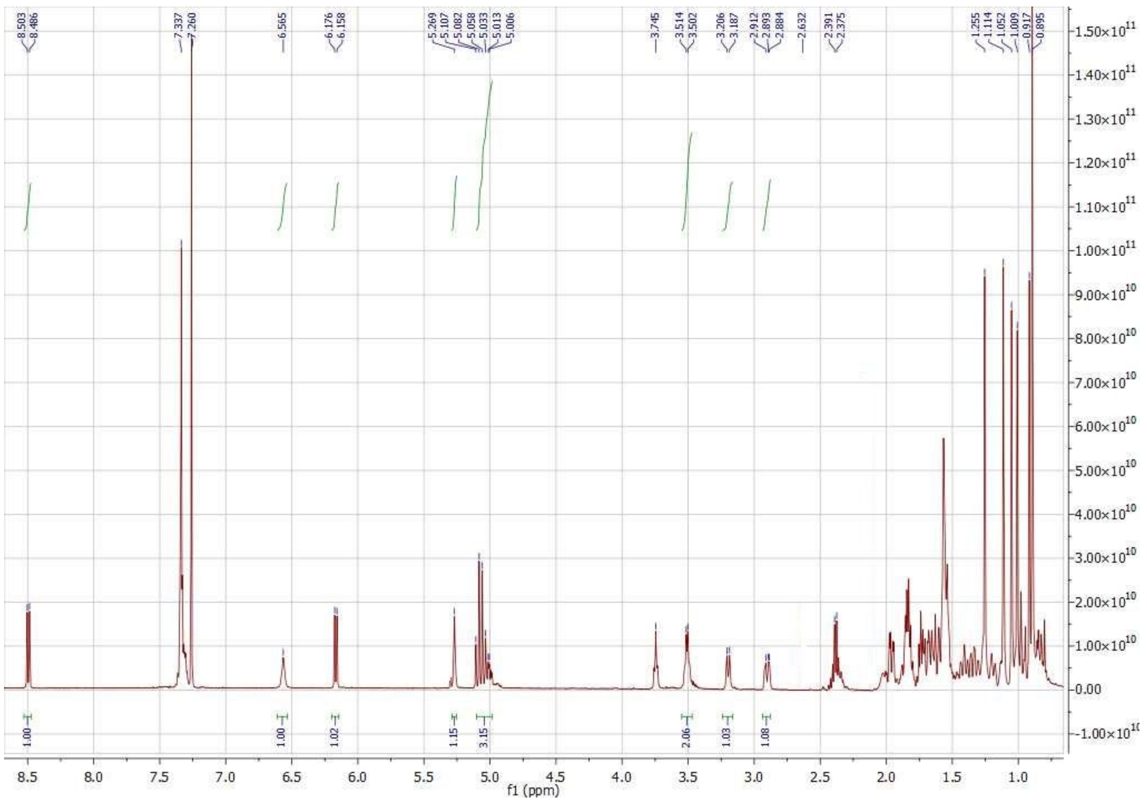

Figure S8: ^{13}C NMR spectrum of compound **4** ($CDCl_3$, 125 MHz).

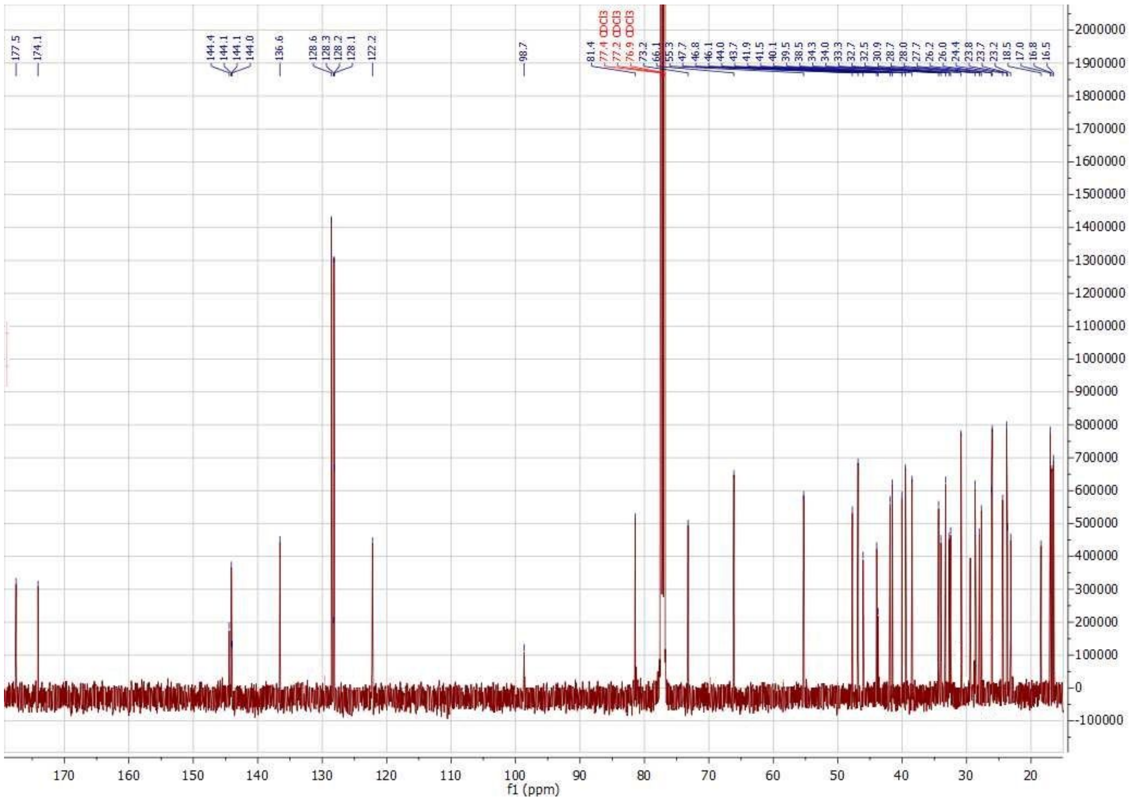

Figure S9: ^{1}H NMR spectrum of compound **5** ($CDCl_3$, 400 MHz).

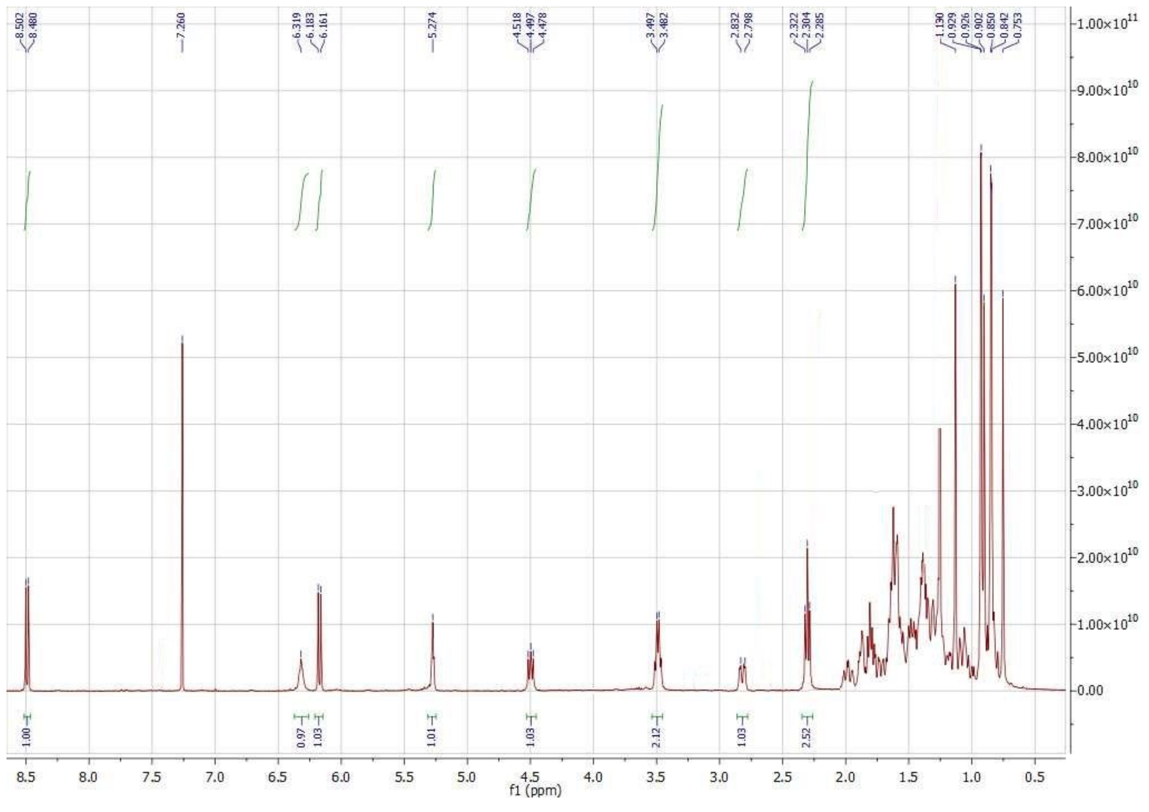

Figure S10: ^{13}C NMR spectrum of compound **5** ($CDCl_3$, 100 MHz).

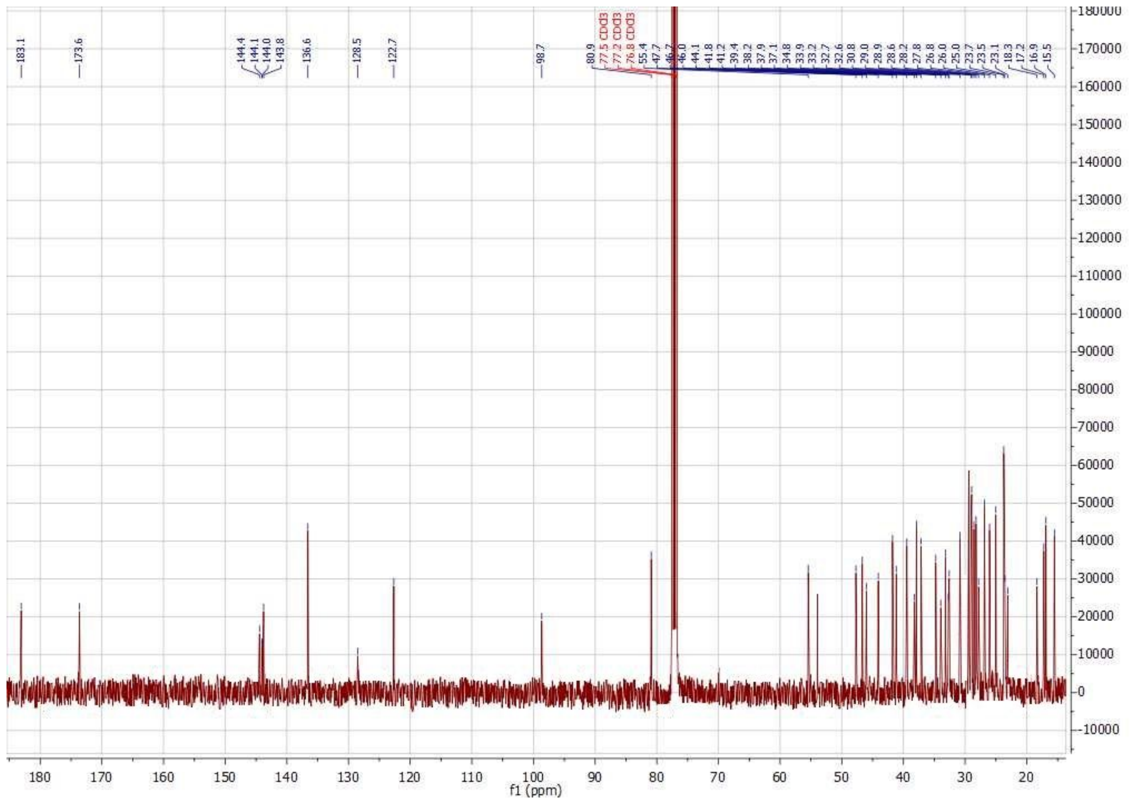

Figure S11: ^{1}H NMR spectrum of compound **6** ($CDCl_3$, 400 MHz).

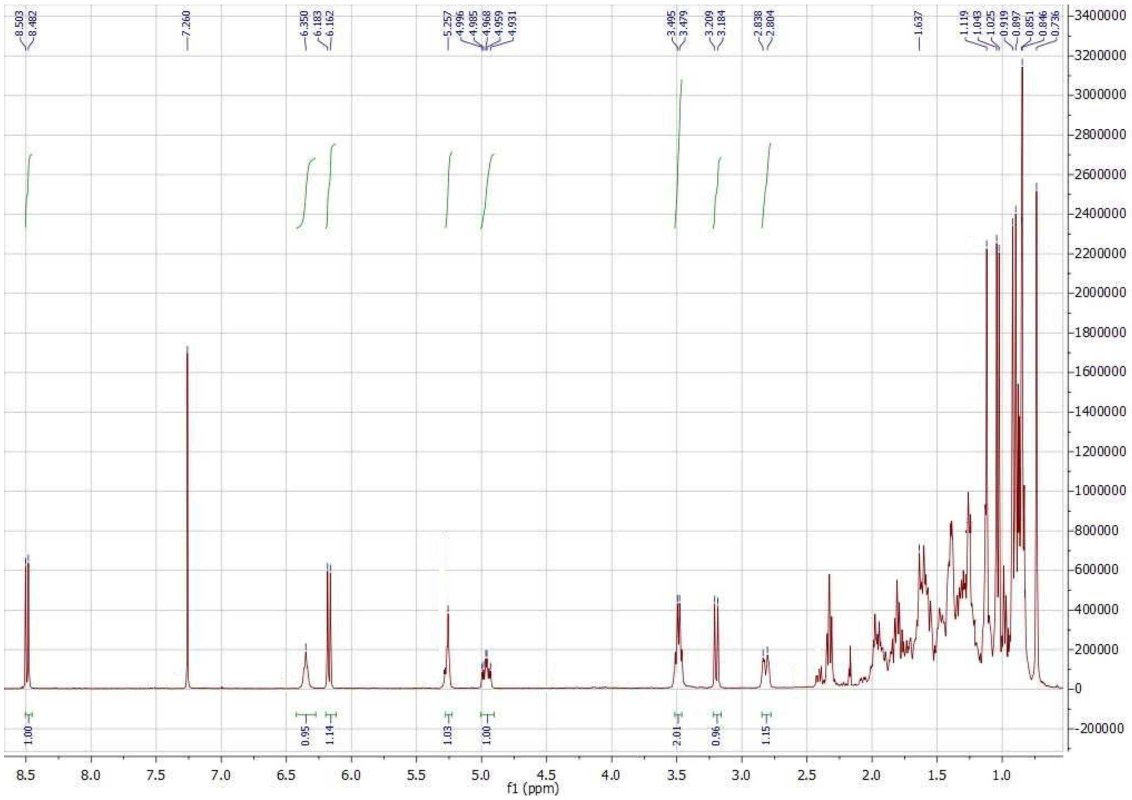

Figure S12: ^{13}C NMR spectrum of compound **6** ($CDCl_3$, 100 MHz).

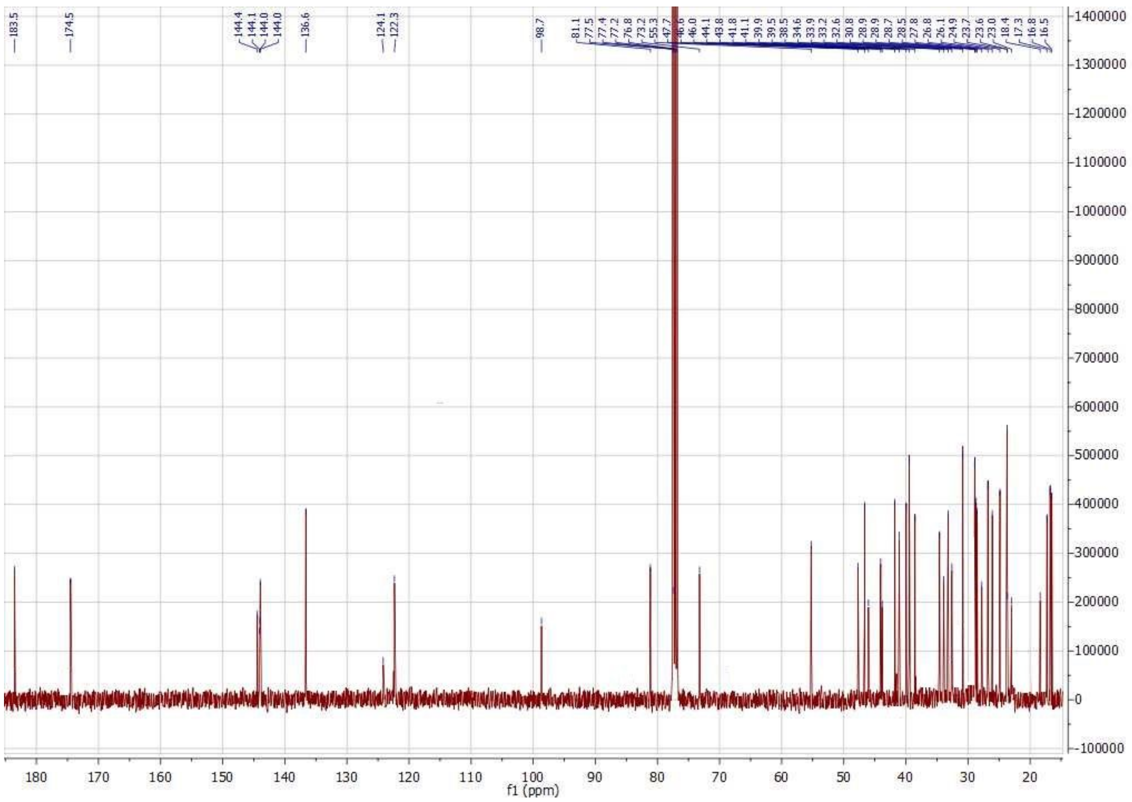

Figure S13: ^{1}H NMR spectrum of compound **7** ($CDCl_3$, 500 MHz).

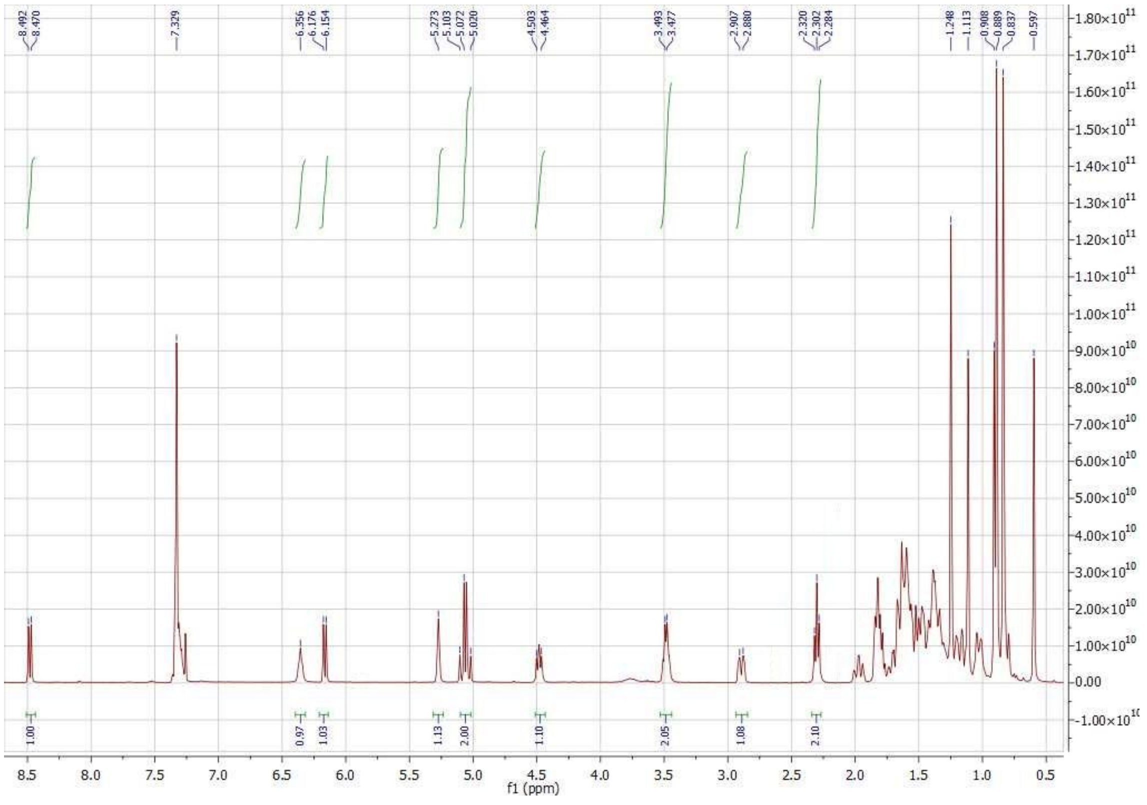

Figure S14: ^{13}C NMR spectrum of compound **7** ($CDCl_3$, 125 MHz).

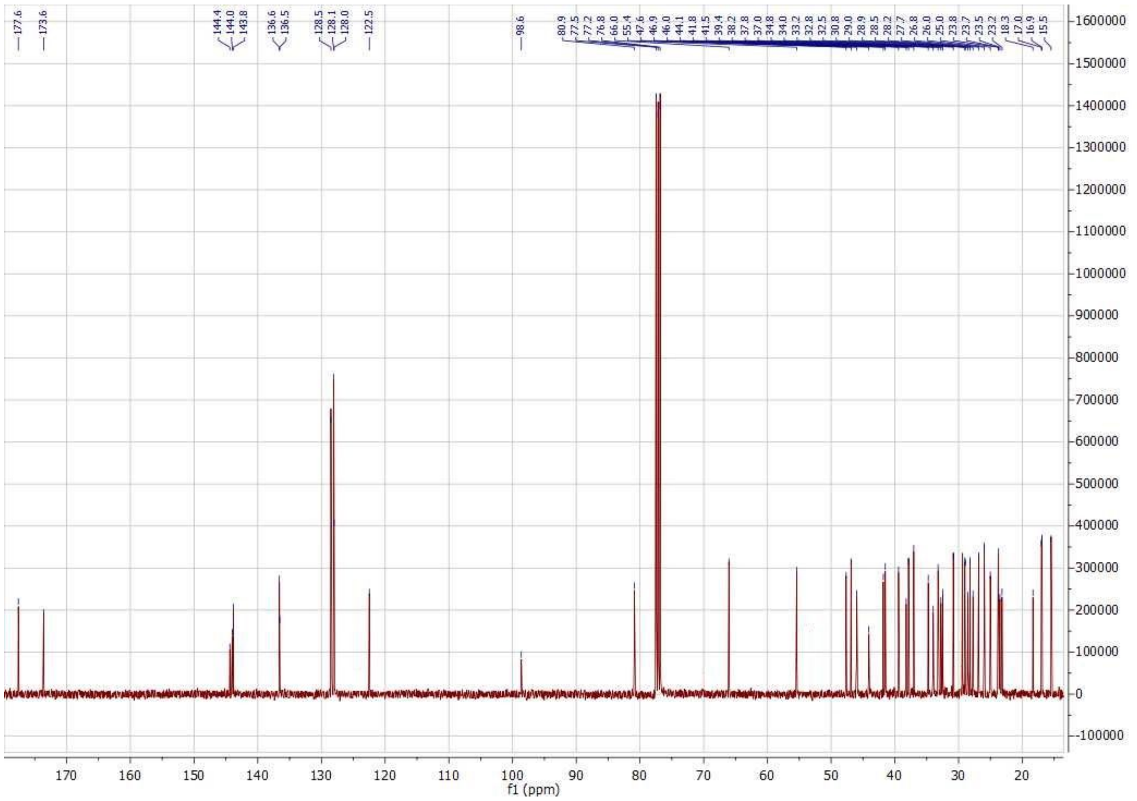

Figure S15: ^{1}H NMR spectrum of compound **8** ($CDCl_3$, 500 MHz).

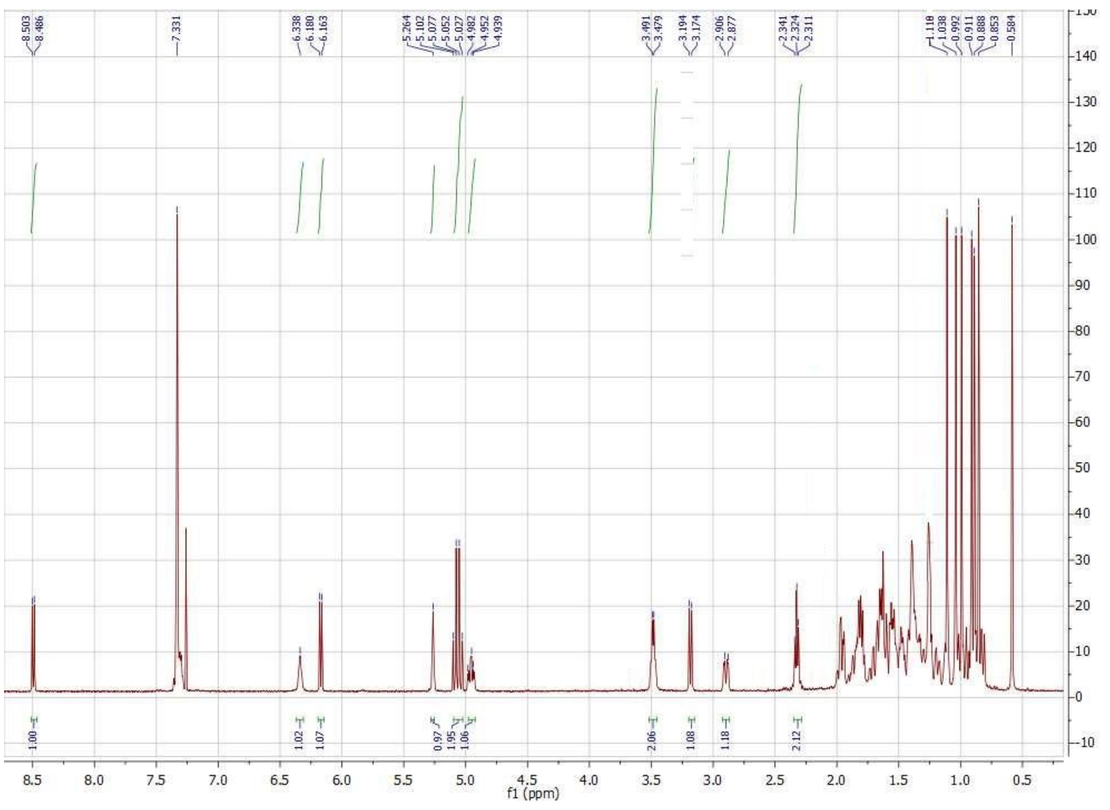

Figure S16: ^{13}C NMR spectrum of compound **8** ($CDCl_3$, 125 MHz).

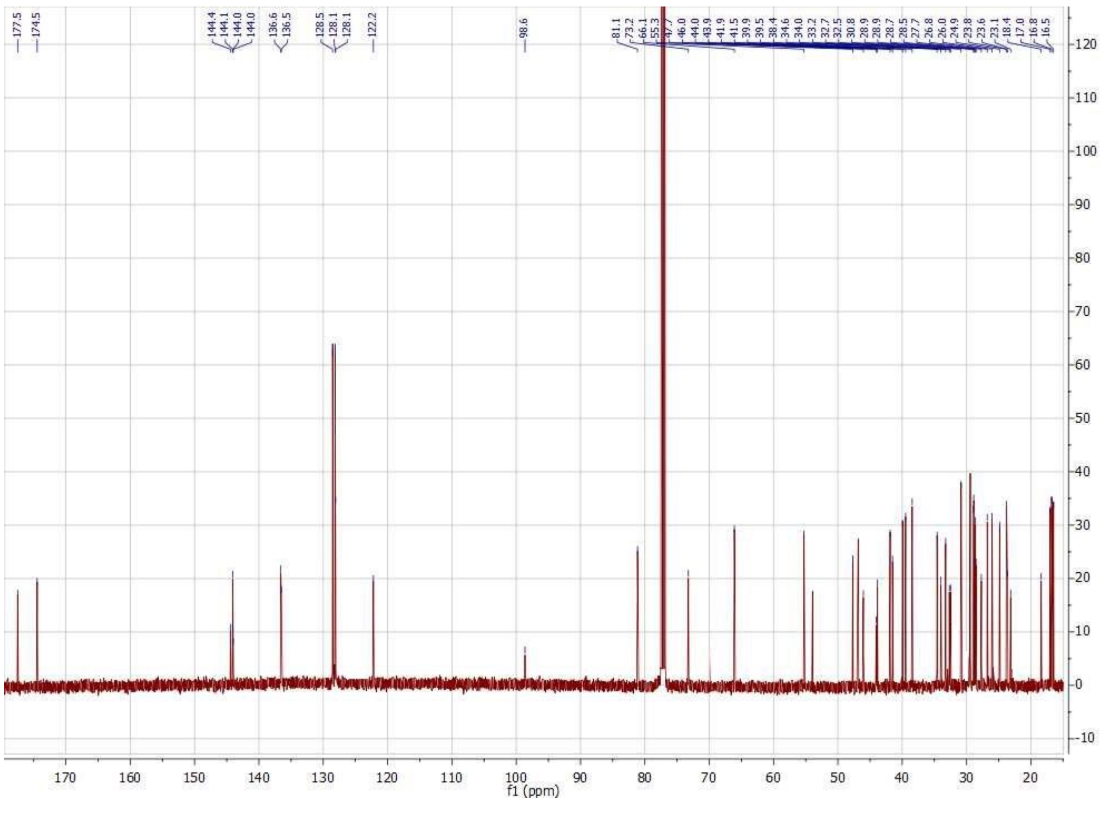

Figure S17: ^{1}H NMR spectrum of compound **9** ($CDCl_3$, 500 MHz).

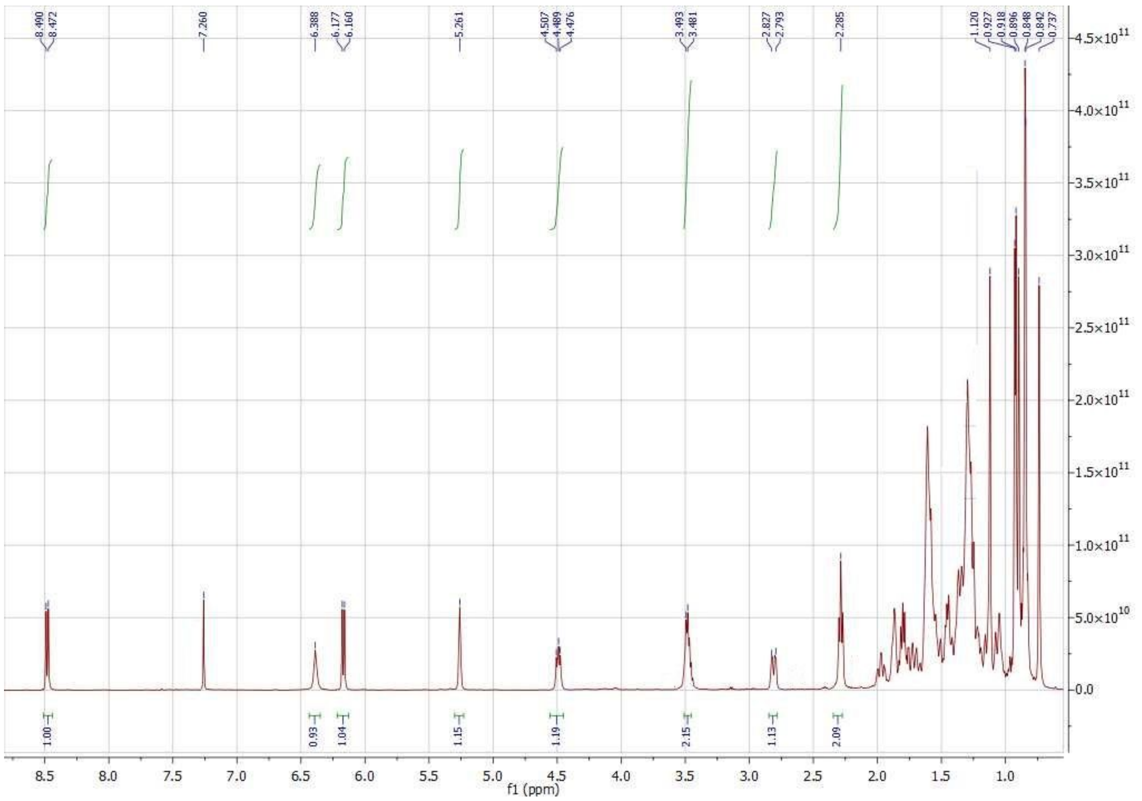

Figure S18: ^{13}C NMR spectrum of compound **9** ($CDCl_3$, 125 MHz).

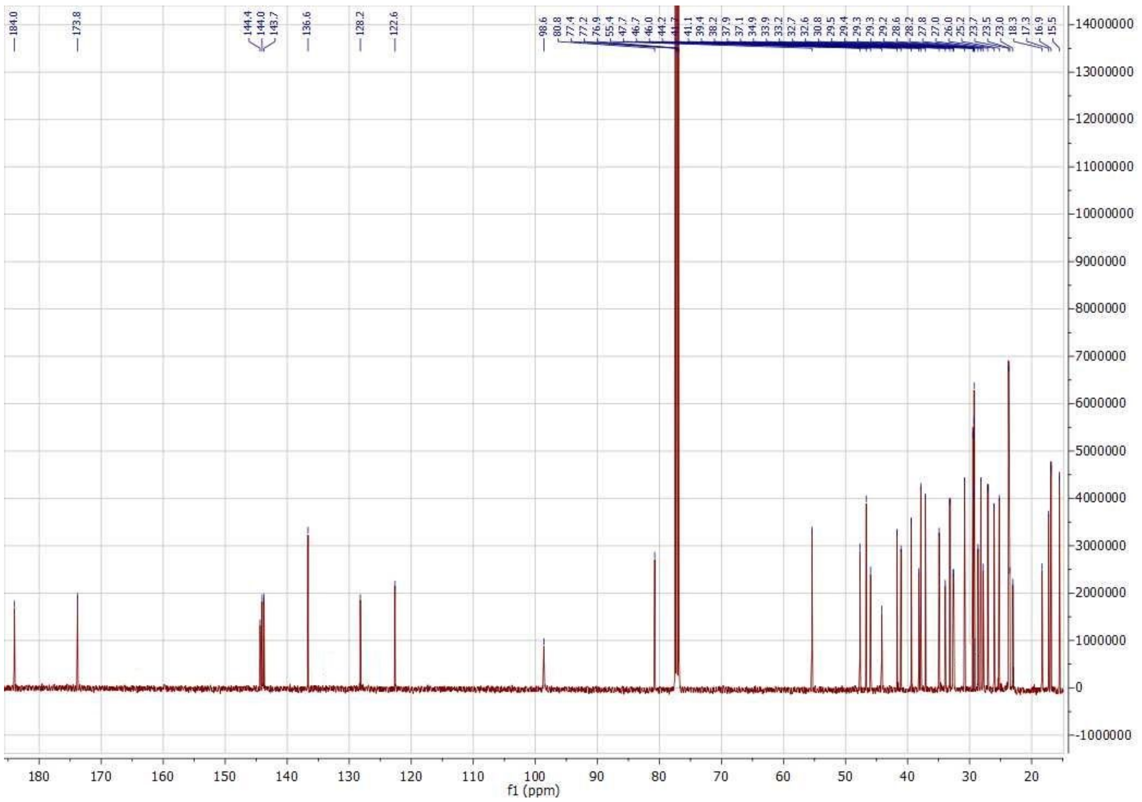

Figure S19: ^{1}H NMR spectrum of compound **10** ($CDCl_3$, 500 MHz).

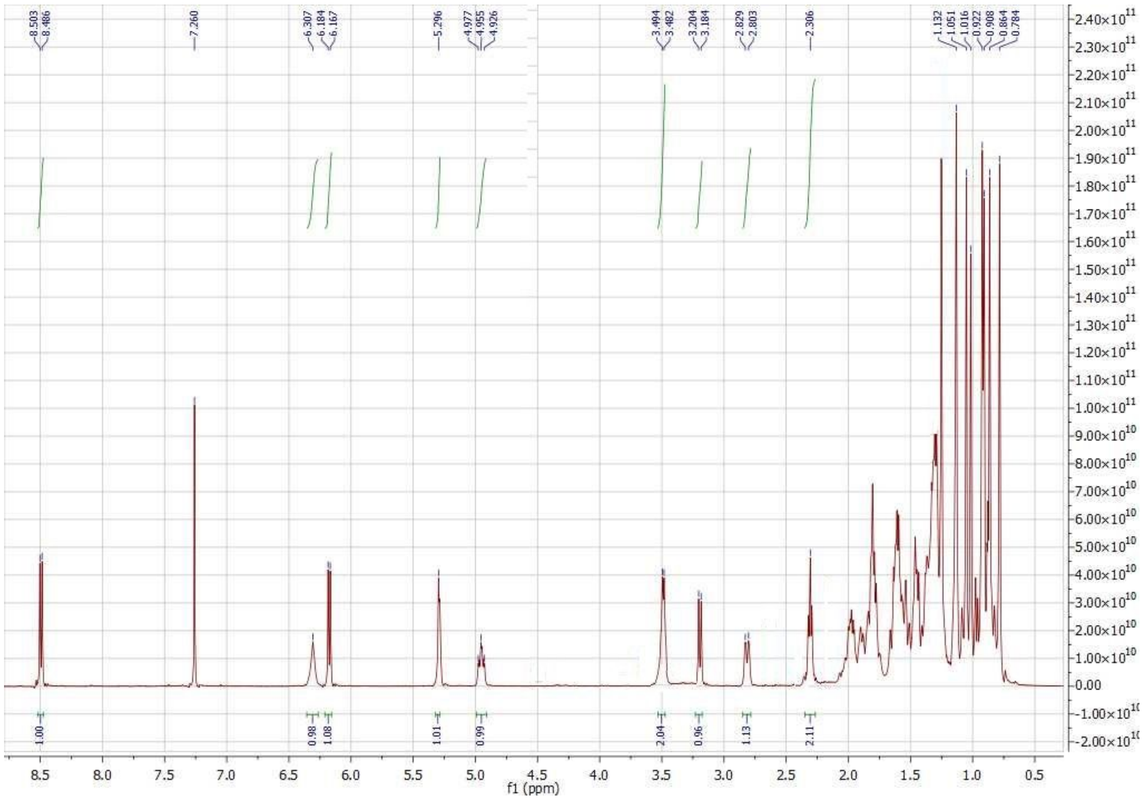

Figure S20: ^{13}C NMR spectrum of compound **10** ($CDCl_3$, 125 MHz).

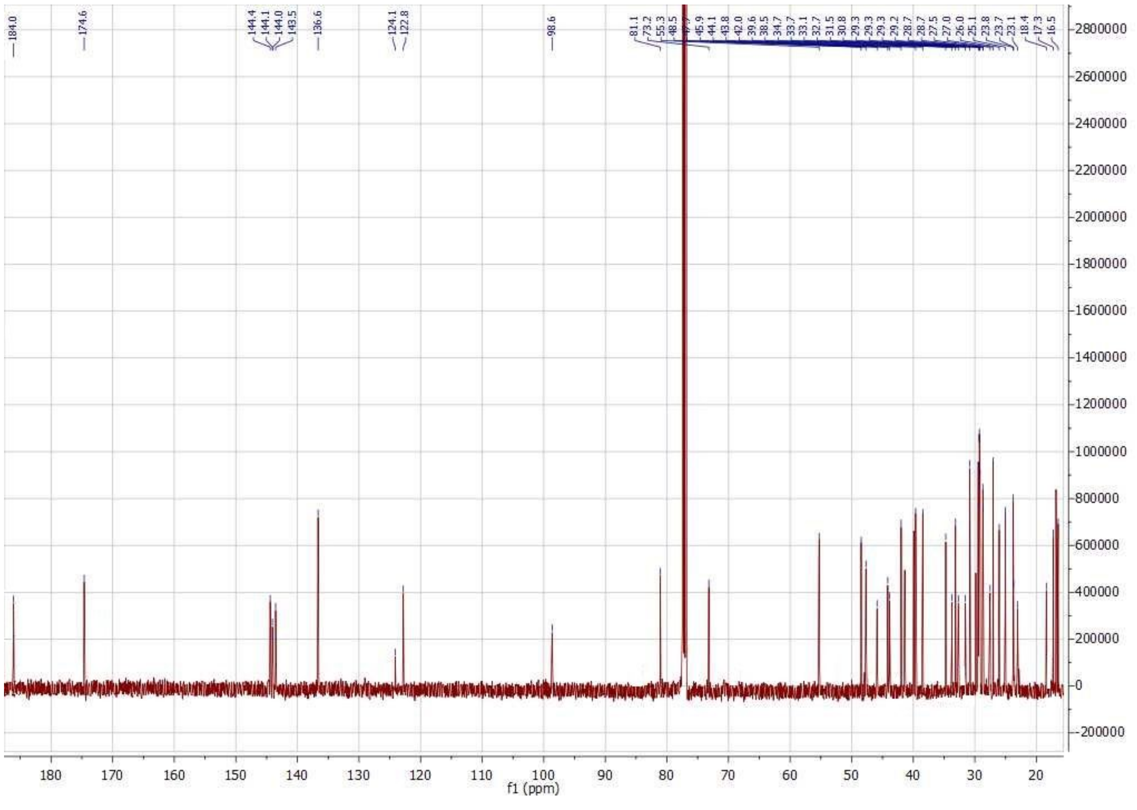

Figure S21: ^{1}H NMR spectrum of compound **11** ($CDCl_3$, 500 MHz).

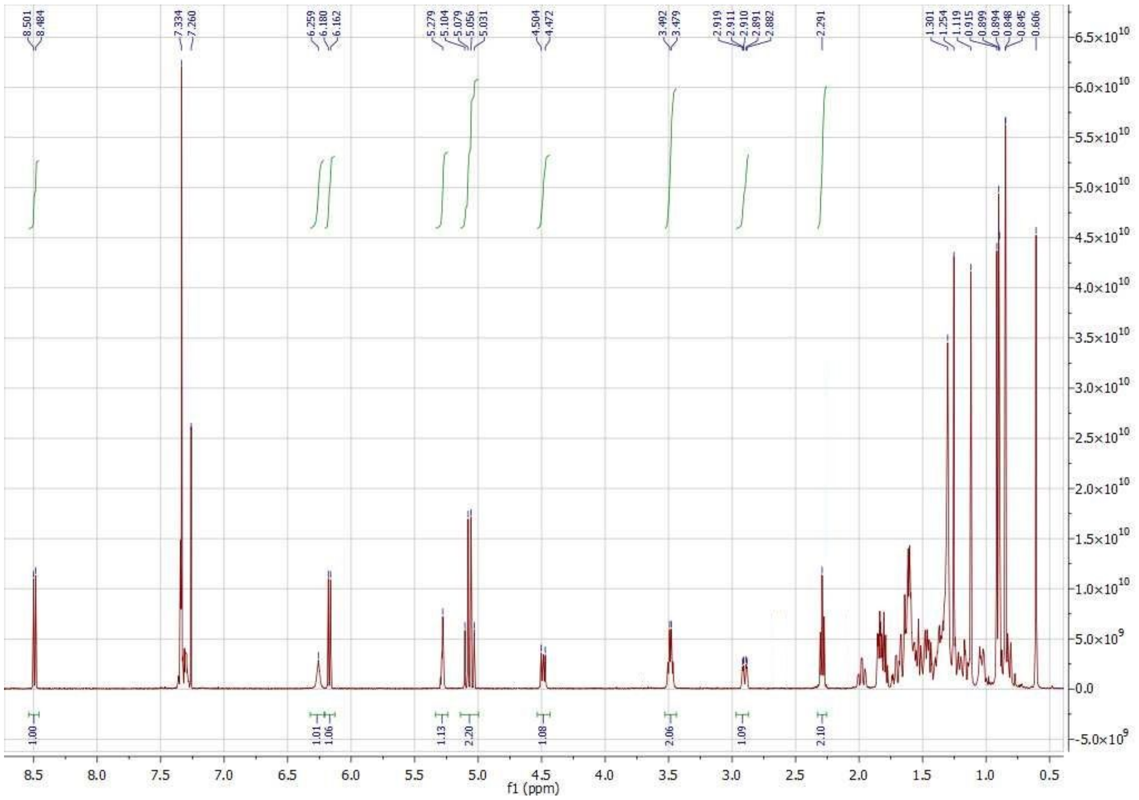

Figure S22: ^{13}C NMR spectrum of compound **11** ($CDCl_3$, 125 MHz).

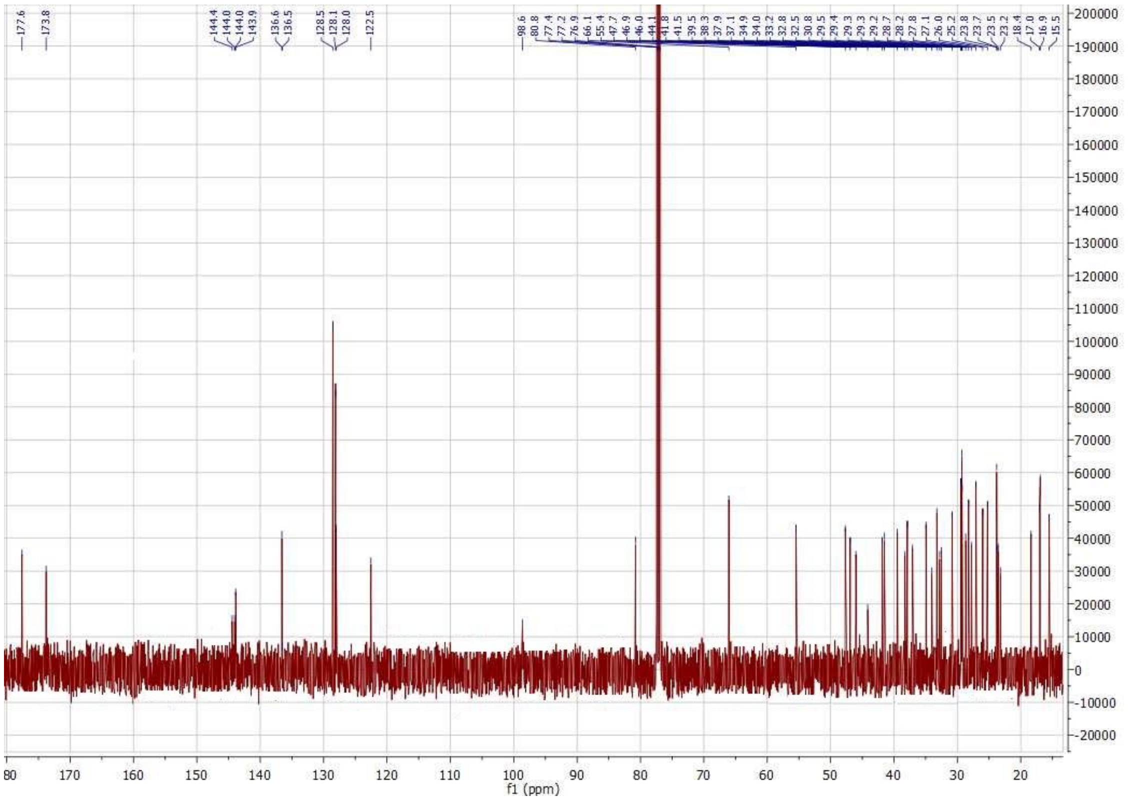

Figure S23: ^{1}H NMR spectrum of compound **12** ($CDCl_3$, 500 MHz).

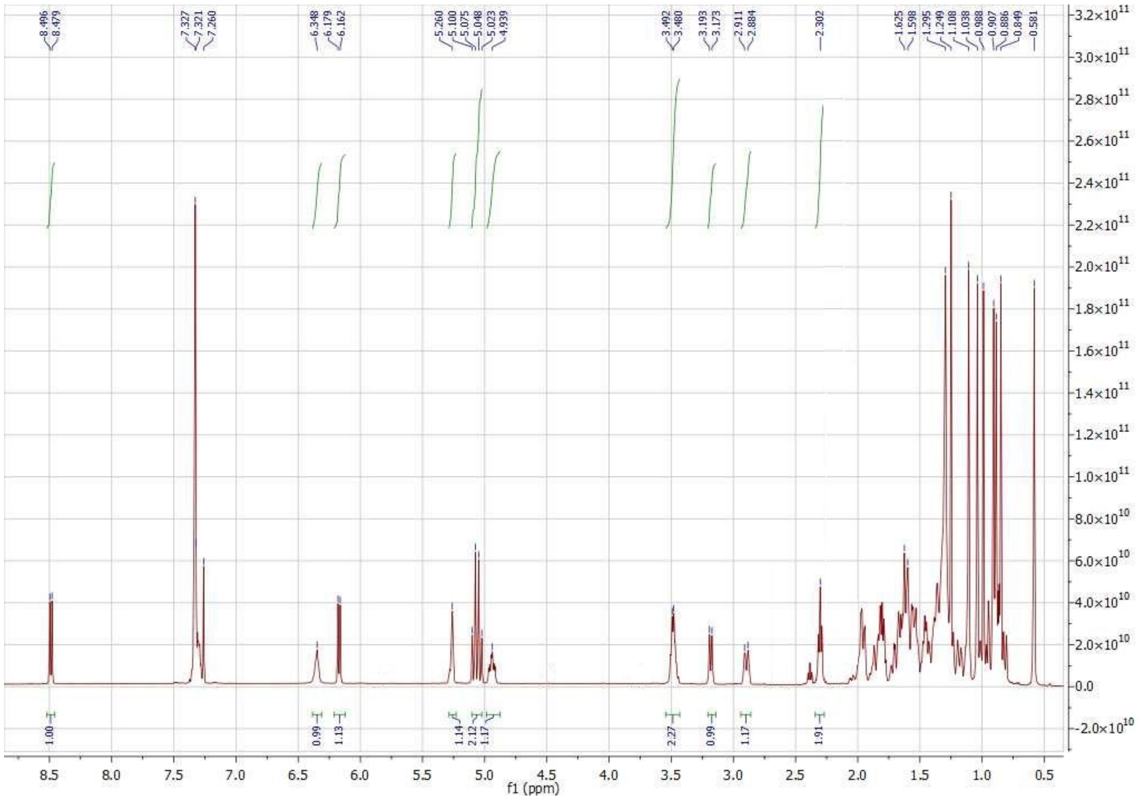

Figure S24: ^{13}C NMR spectrum of compound **12** ($CDCl_3$, 125 MHz).

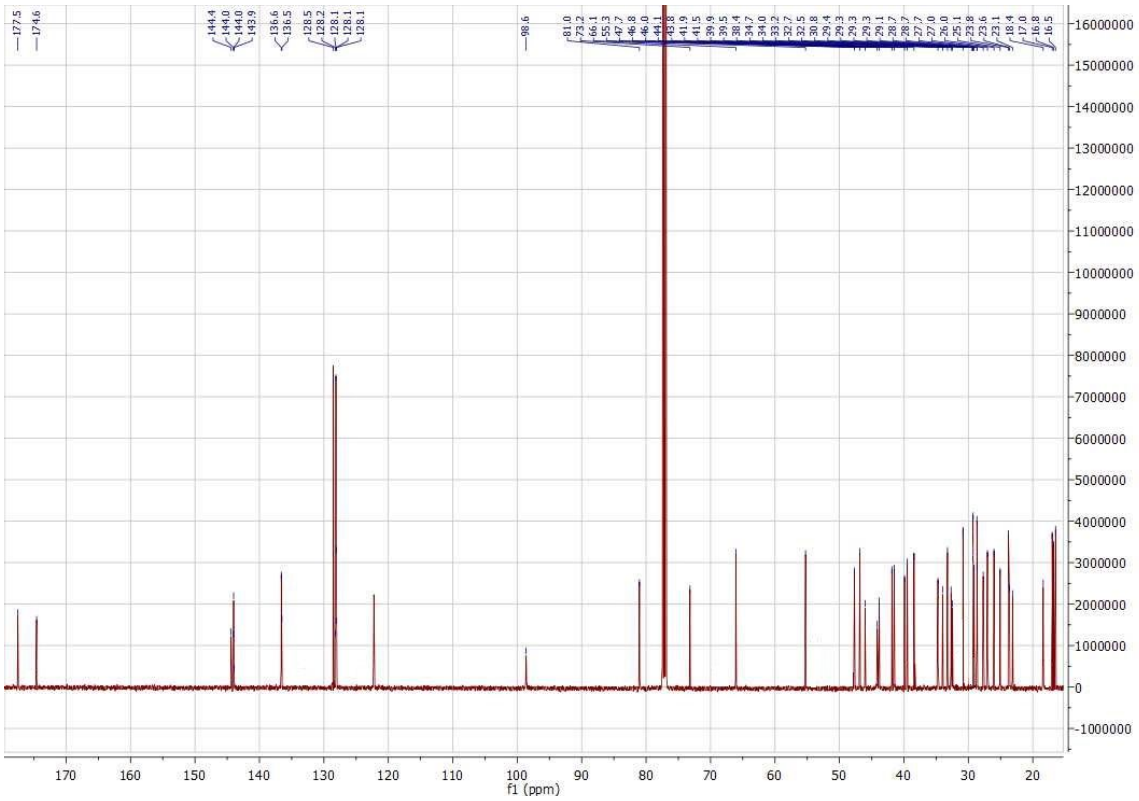

Figure S25: Graphs of cell viability percentages of compound **1** (IC_{50}) in 3 cancer-cell lines

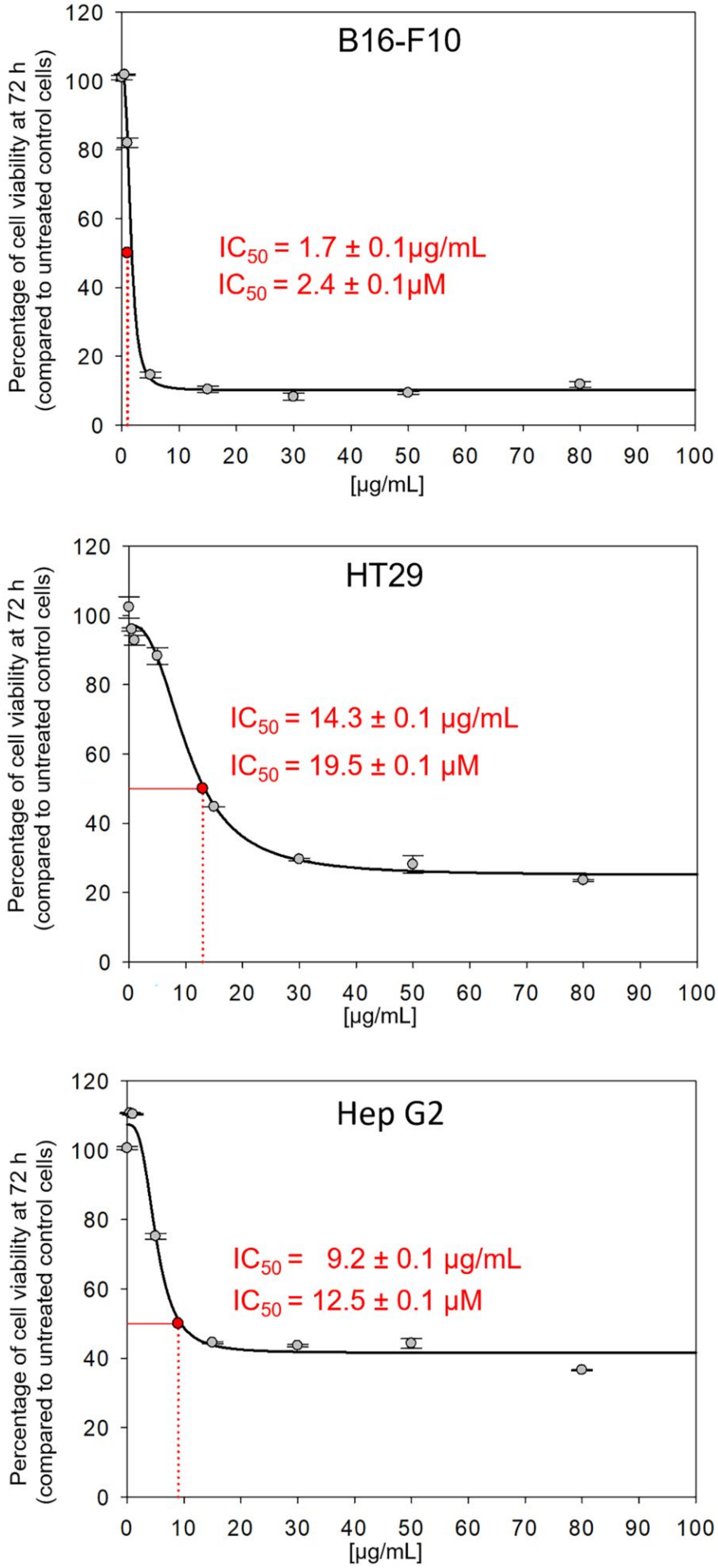

Figure S26: Graphs of cell viability percentages of compound **2** (IC_{50}) in 3 cancer-cell lines

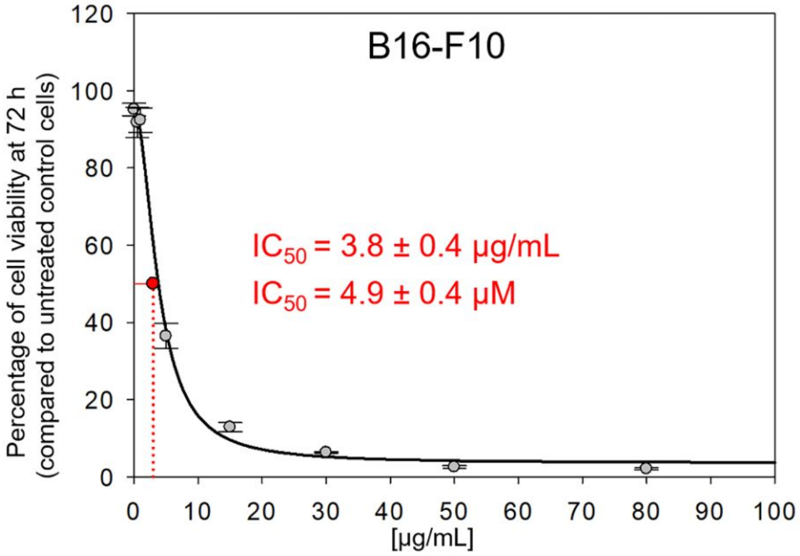

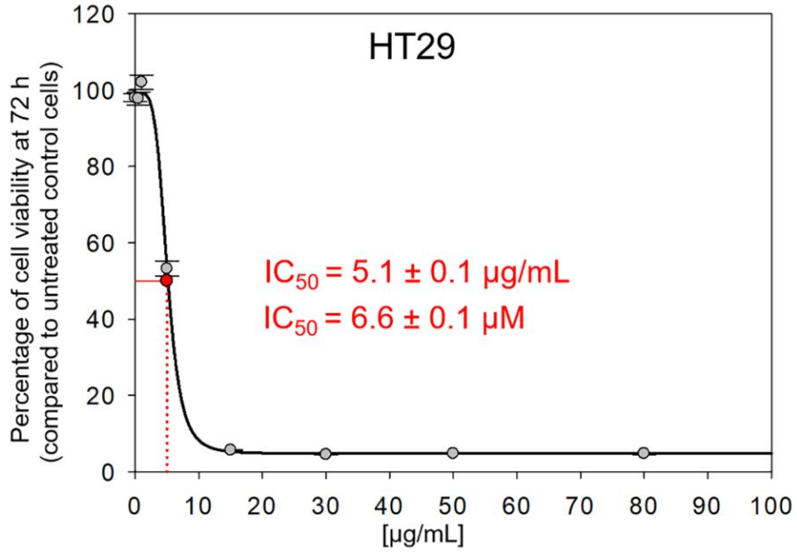

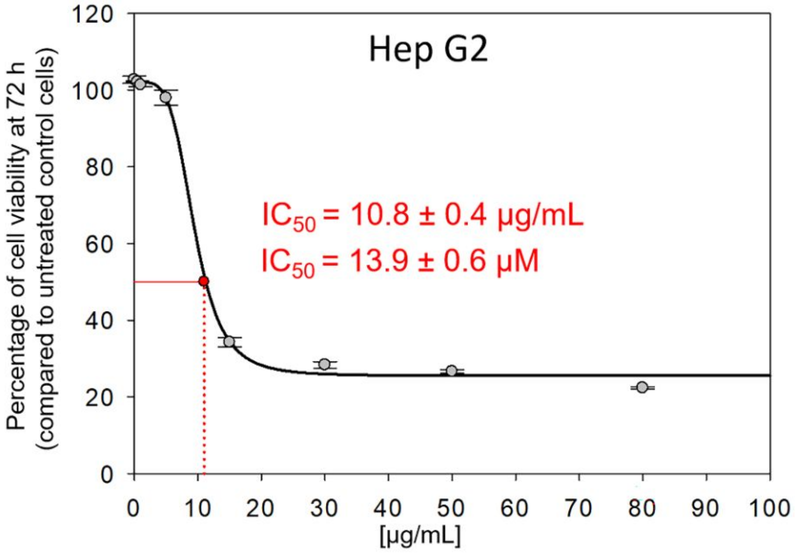

Figure S27: Graphs of cell viability percentages of compound **3** (IC_{50}) in 3 cancer-cell lines

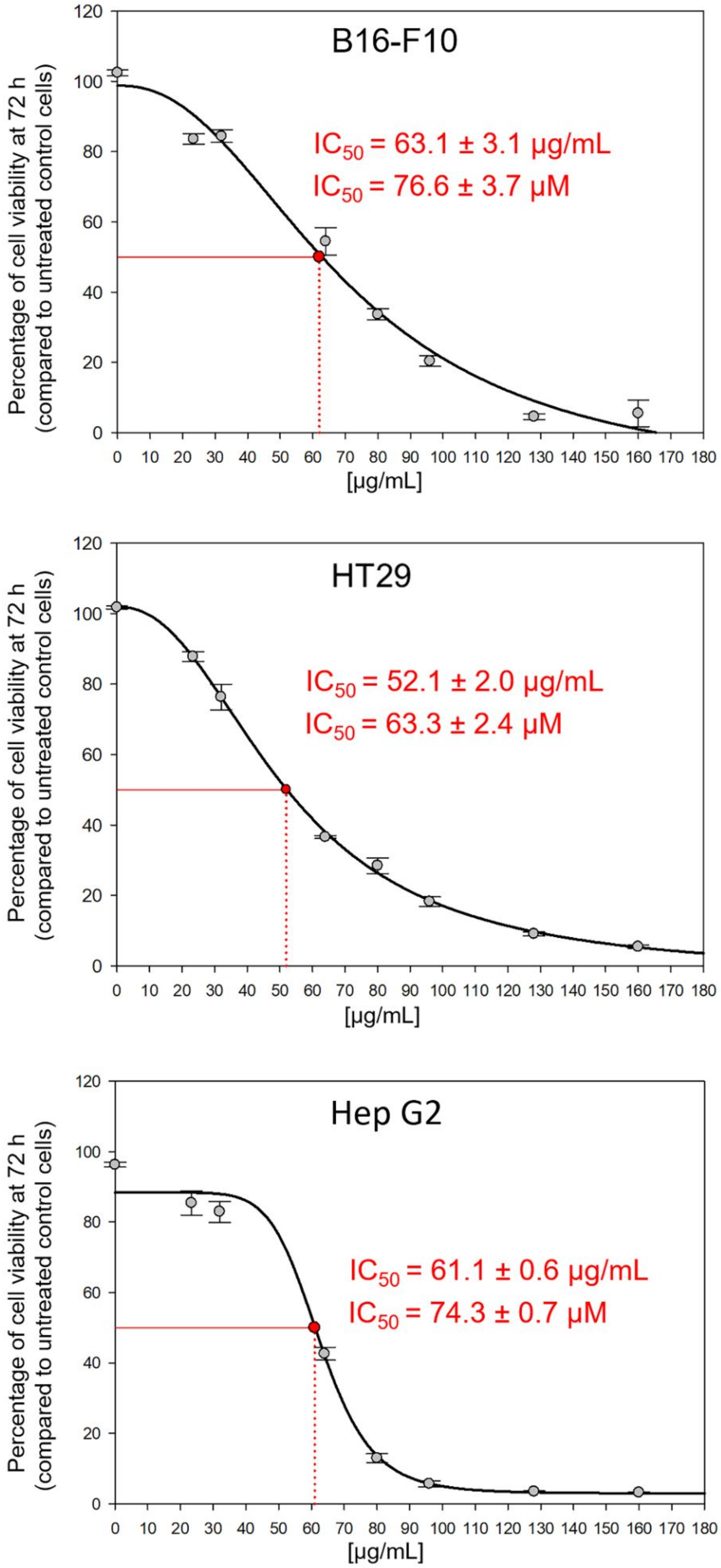

Figure S28: Graphs of cell viability percentages of compound **4** (IC_{50}) in 3 cancer-cell lines

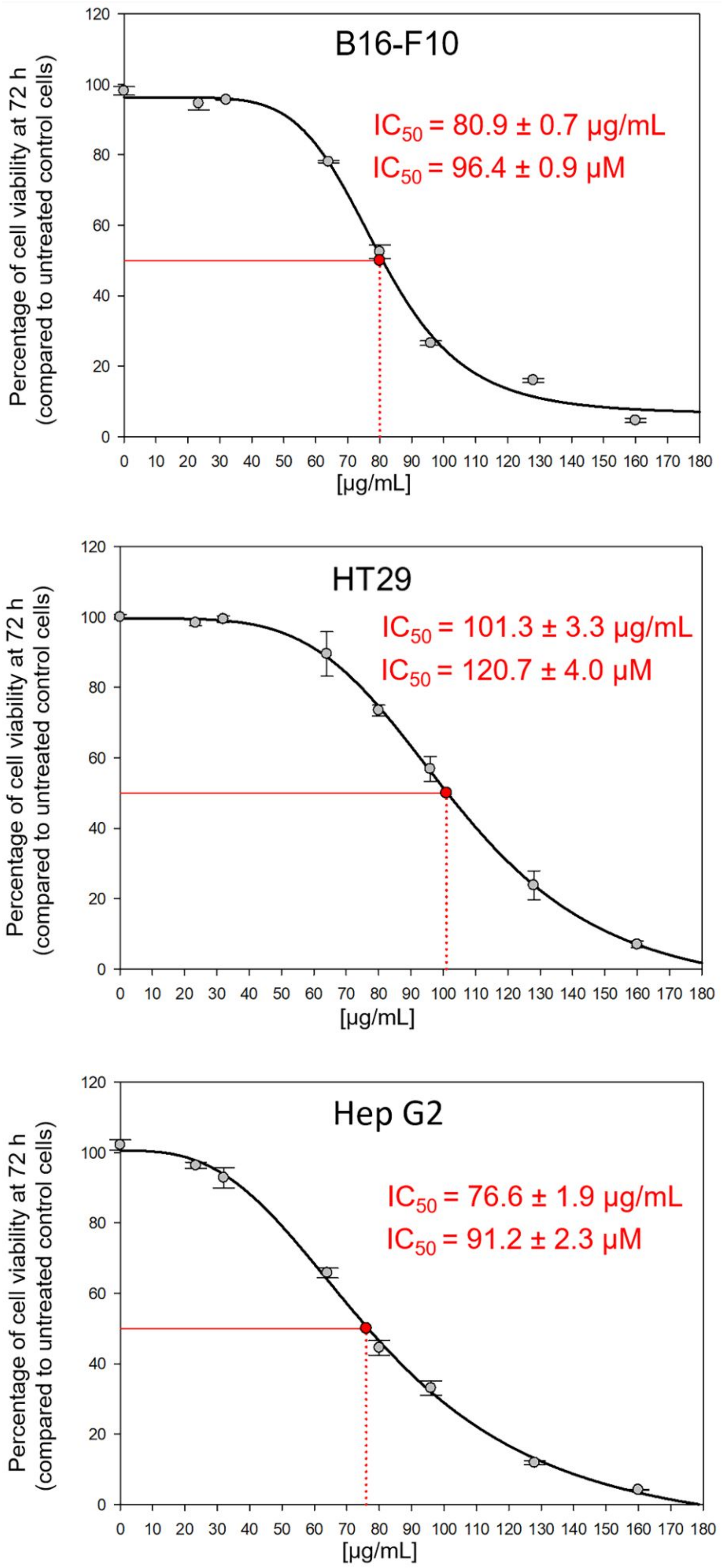

Figure S29: Graphs of cell viability percentages of compound **5** (IC_{50}) in 3 cancer-cell lines

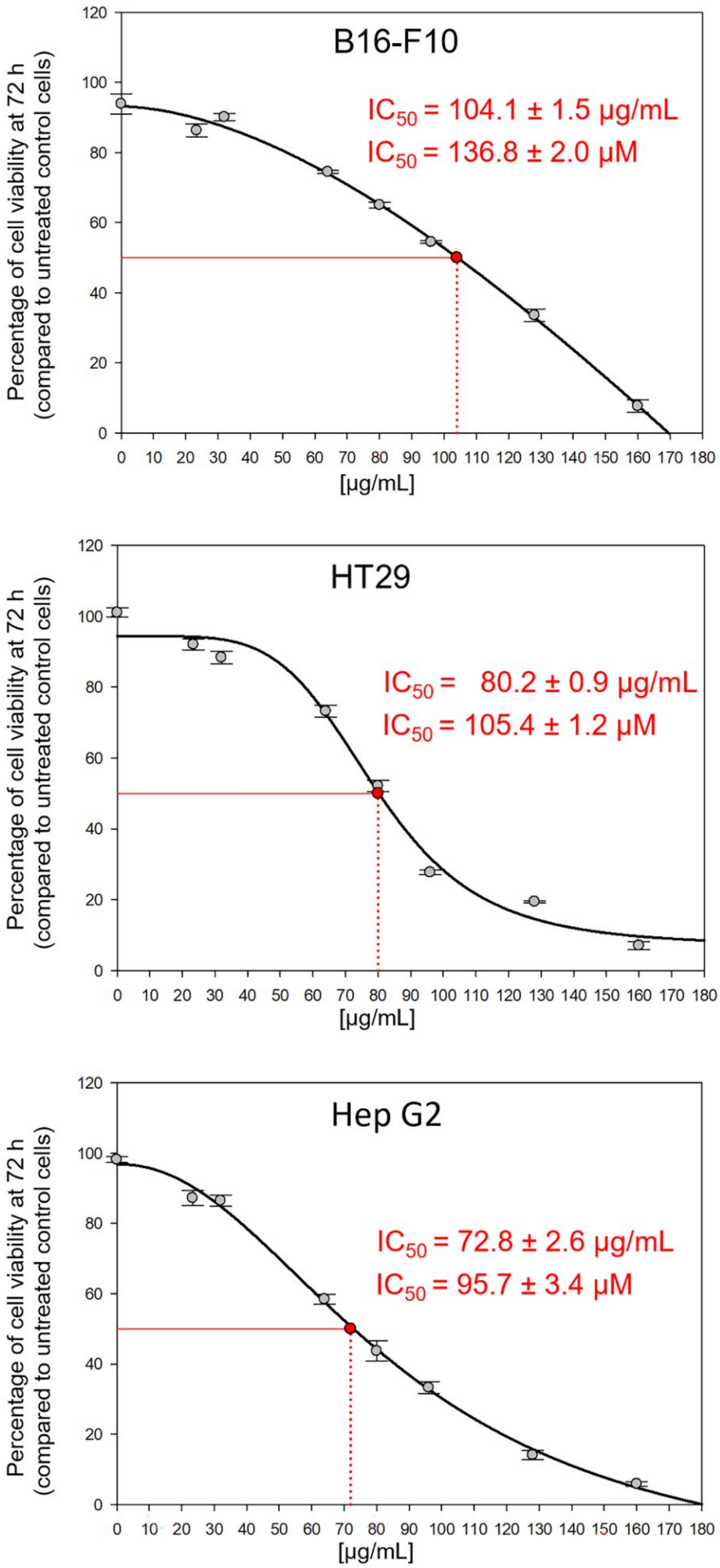

Figure S30: Graphs of cell viability percentages of compound **6** (IC_{50}) in 3 cancer-cell lines

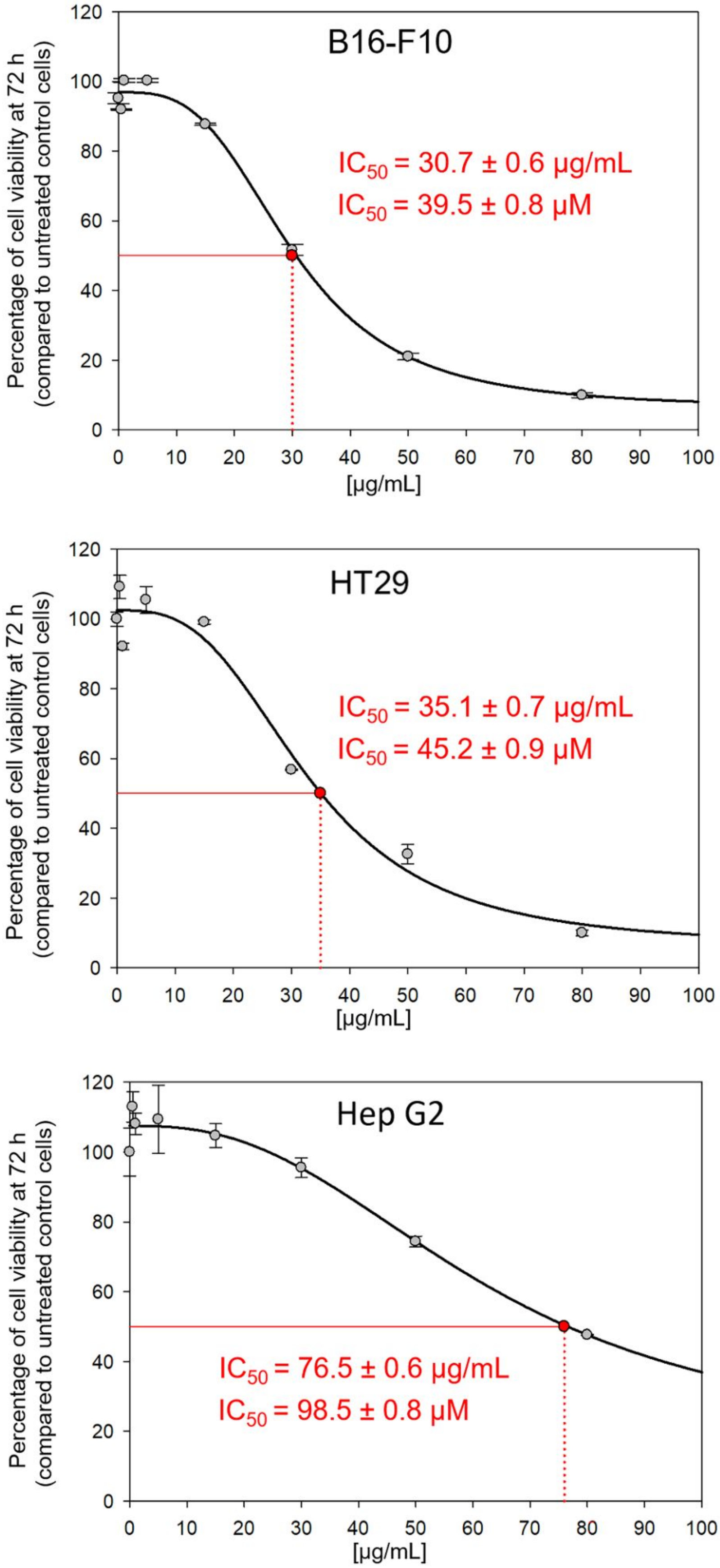

Figure S31: Graphs of cell viability percentages of compound **7** (IC_{50}) in 3 cancer-cell lines

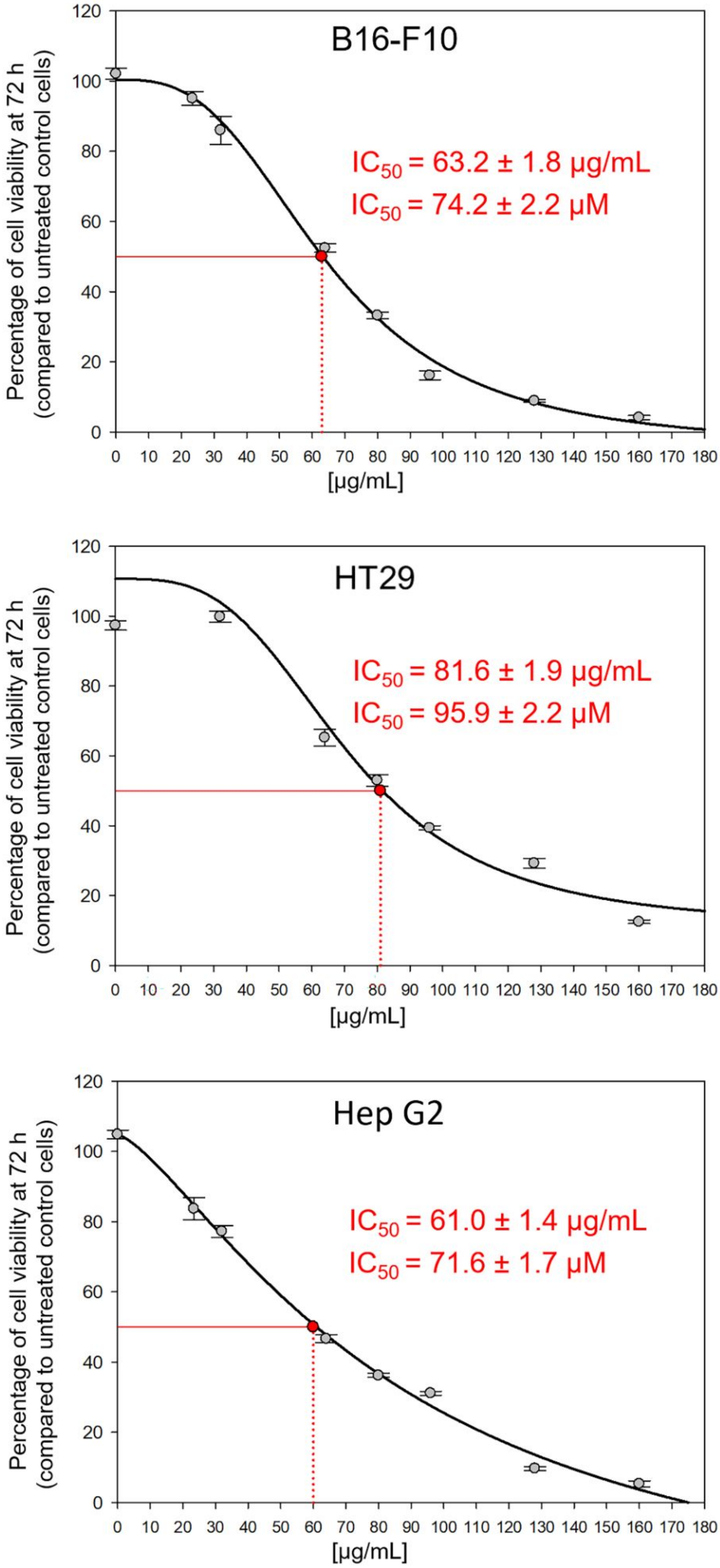

Figure S32: Graphs of cell viability percentages of compound **8** (IC_{50}) in 3 cancer-cell lines

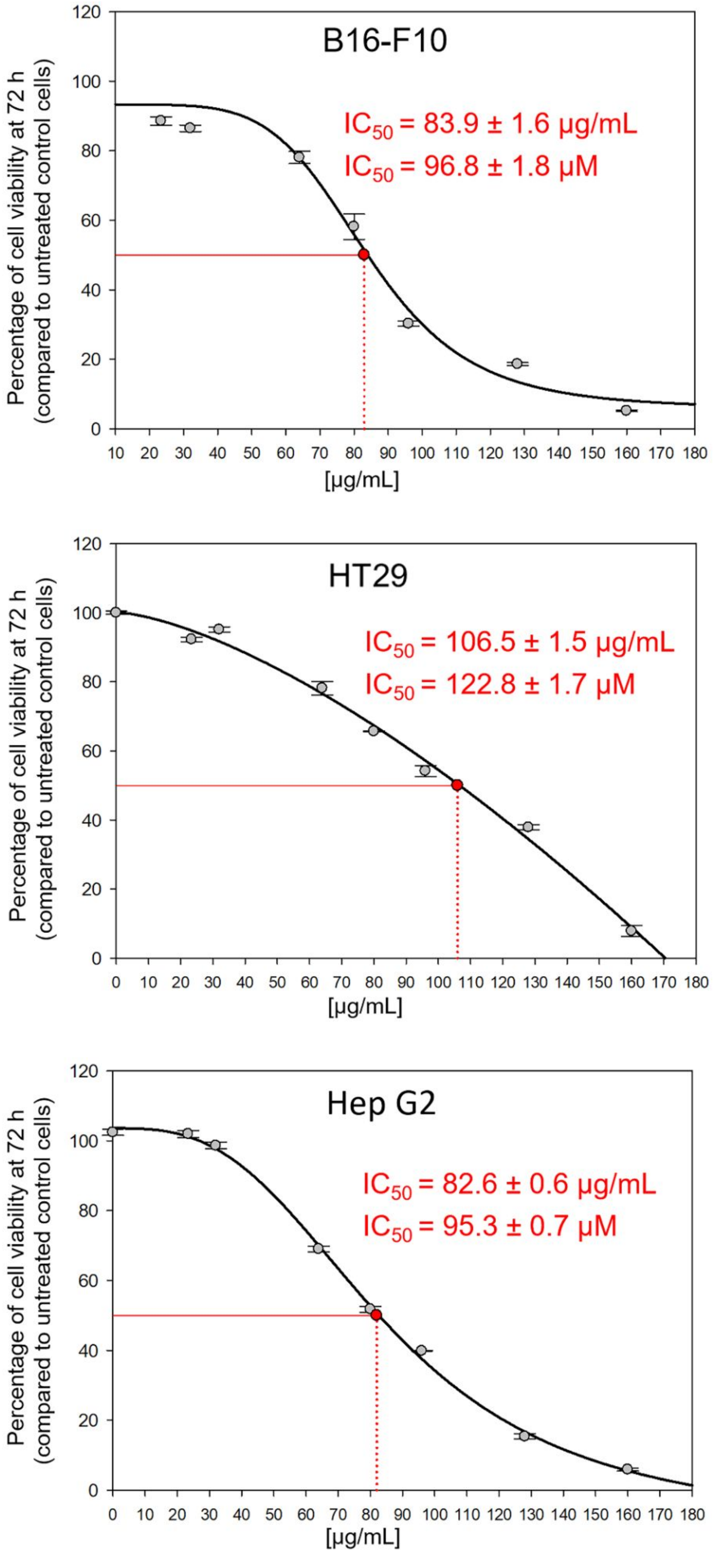

Figure S33: Graphs of cell viability percentages of compound **9** (IC_{50}) in 3 cancer-cell lines

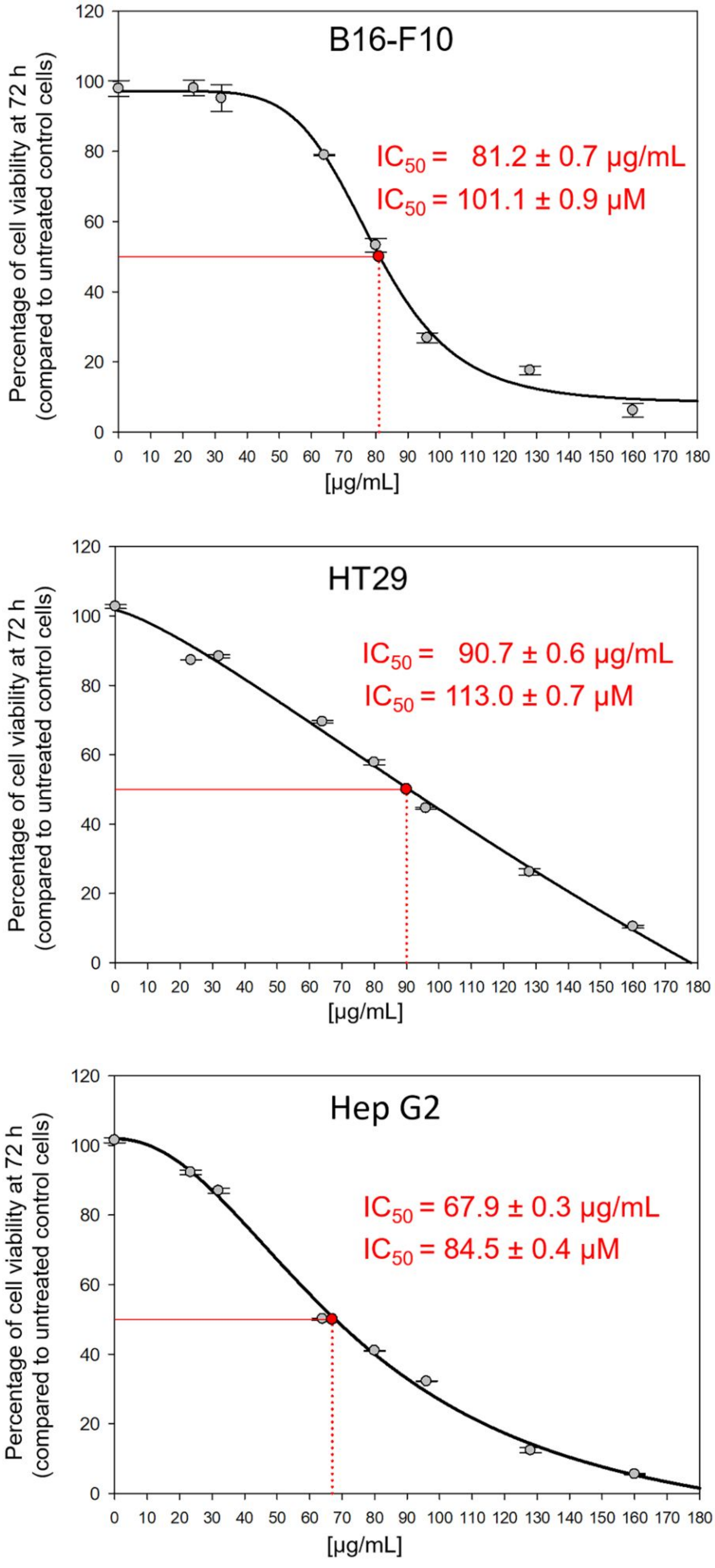

Figure S34: Graphs of cell viability percentages of compound **10** (IC_{50}) in 3 cancer-cell lines

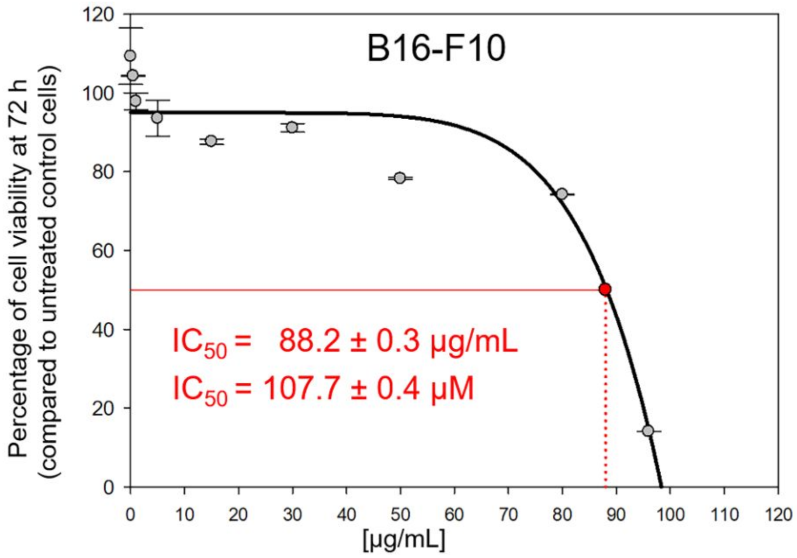

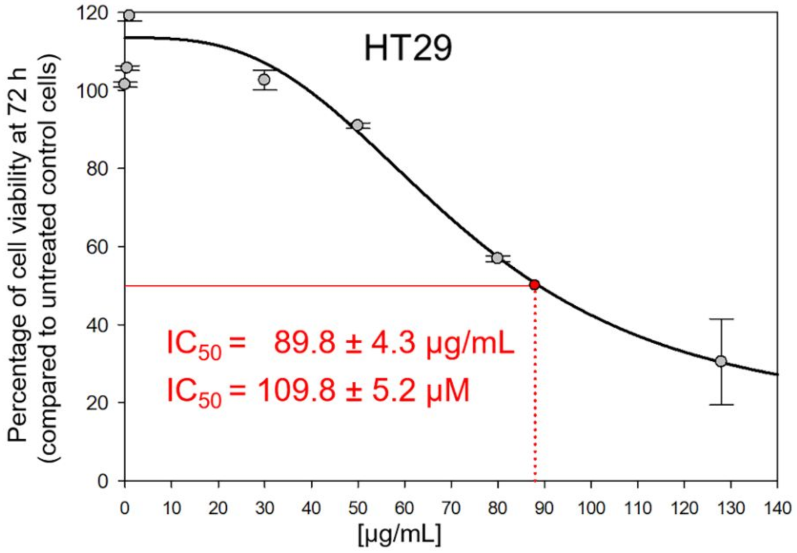

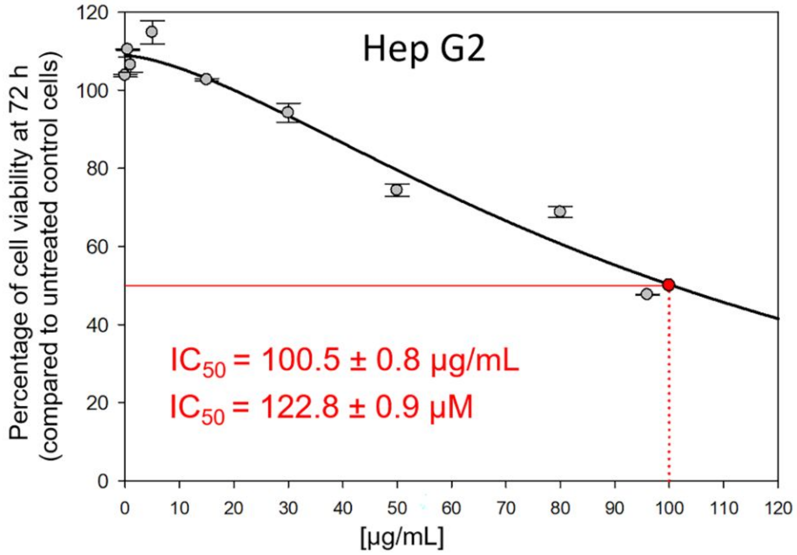

Figure S35: Graphs of cell viability percentages of compound **11** (IC_{50}) in 3 cancer-cell lines

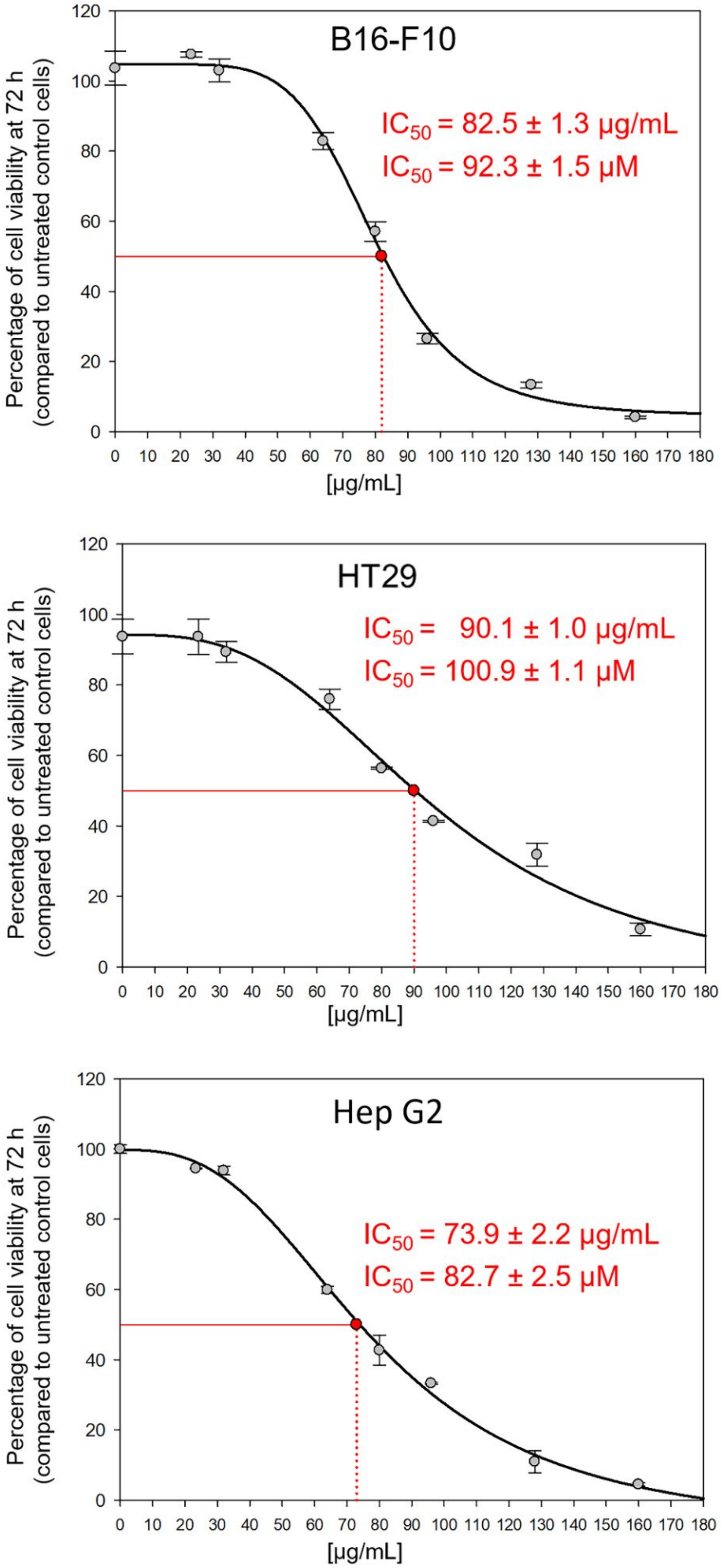

Figure S36: Graphs of cell viability percentages of compound **12** (IC_{50}) in 3 cancer-cell lines

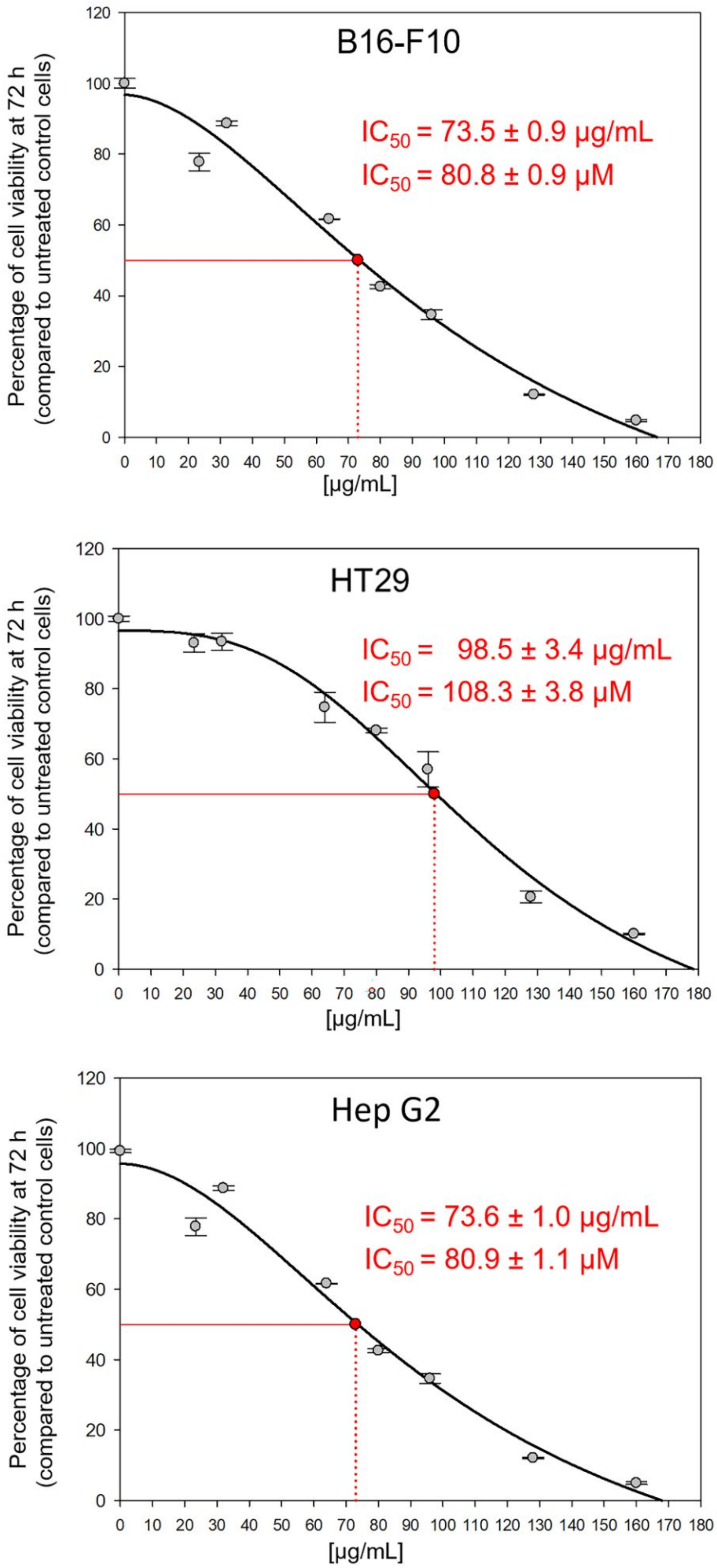

Supplement: Supplementary file 1 — np2c00880_si_001.pdf [file np2c00880_si_001.pdf]
